# Molecular Origins of Philicity: How Atomic Interactions Determine Miscibility and Diffusivity

**DOI:** 10.1002/cphc.202500875

**Published:** 2026-04-25

**Authors:** Anna Luisa Upterworth, Tom Erik Steinkopf, Daniel Sebastiani

**Affiliations:** ^1^ Institute of Chemistry Martin Luther University Halle‐Wittenberg Halle Germany

**Keywords:** entropy of mixing, miscibility, molecular dynamics, polyphilicity

## Abstract

We present a computational study on the microscopic origin of molecular philicity, which determines the miscibility and diffusivity of binary liquids. Our simulations reveal how the miscibility of an alkane‐perfluoroalkane system responds to variations in the philicity of the two components, which is controlled by tuning the Lennard‐Jones force‐field parameters. One particular challenge is measuring the instantaneous degree of mixing of the two components. Here, we use two criteria: one based on the pair correlation function and one based on the instantaneous configurational entropy of mixing. We observe a characteristic linear dependence of the critical temperature of mixing on the interaction energy values. The critical temperature is considerably more sensitive to changes in the aliphatic alkanes than in the fluorinated ones. In contrast, the size parameter has a negligible influence on either molecule.

## Graphical abstract

Alterations in the atomic philicity, as represented by Lennard–Jones parameters, determine the miscibility of an alkane‐perfluoroalkane system. Some parameters enhance mixing, while others enhance phase separation.

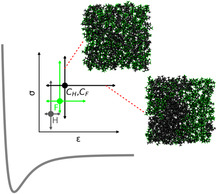

## 1 Introduction

In the field of molecular liquids, there is the concept of philicity as a property of molecules that determines their affinity toward molecules of the same species versus molecules of other species with “different” philicity. As it is a qualitative concept, there is no single measurable parameter that represents this molecular property in its full functionality. Hence, several terms have evolved in literature that describe this concept. The most popular variant is known as hydrophilicity, but other common ones include lipophilicity, fluorophilicity, and silanophilicity [1, 2, 3, 4]. Note that there are also “false friends,” such as metallophilicity, which is a noncovalent interaction of heavy metal atoms of a similar strength to a hydrogen bond, and aurophilicity, which is a particular type of metallophilicity. In this context, the term “philicity” refers to a specific short‐range, directional atom–atom interaction, but not to the miscibility of two different molecular species. In this work, we focus on the latter meaning from a theoretical perspective, i.e., we aim to shed light on the underlying atomic interactions that determine whether the philicities of two constituents “match” or “mismatch” with regard to their miscibility at a given temperature.

Molecules whose philicity is incompatible with a certain type of species are often said to be “phobic” toward that species. Alkanes, for instance, are hydrophobic and do not mix with water. However, this nomenclature is somewhat problematic, since the term “phobicity” implies repulsion, even though there is, of course, an attractive net interaction between water and alkane molecules. The “philicity mismatch” and immiscibility result from the different strengths of the attractive intermolecular interactions. Since the attraction is considerably weaker for alkane‐water interactions than for the water–water and alkane–alkane interactions, the two species tend to phase separate at low temperatures. Although philicity mainly involves enthalpic effects, it is important to recognize that it is in permanent competition with entropic forces that drive the system toward a mixed state. At high temperatures, the entropic contribution to the free energy outweighs the enthalpic contribution, and the two phases with different philicities become miscible. The state of mixing of a system at a given temperature is determined by the Gibbs free energy:

(1) ΔG=ΔH−TΔS

where ΔH is the enthalpy of mixing (typically positive for philicity‐driven demixing), ΔS is the entropy of mixing (positive due to increased configurational freedom), T is the absolute temperature, and Δ denotes the difference between a fully mixed state and a fully separated state.

However, obtaining the entropic and enthalpic contributions based on an actual simulation is not entirely straightforward. While the configurational entropy of mixing in an ideal perfect mixture is trivial, $\Delta S=-R\sum_{i}x_{i}\ln\;(x_{i})$ (where $x_{i}$ is the molar fraction of component i and R the universal gas constant), its evaluation in a non‐ideal, partially mixed system is not trivial [5, 6, 7, 8, 9, 10]. The same applies to the enthalpy. While the basic interatomic interactions are known, as they are the essential starting point for any simulation, generally in the form of an atomic force field, their species‐averaged configuration‐dependent contributions to the total energy are more difficult to compute [11, 12]. A more specific quantity to consider is the miscibility temperature, i.e., the temperature above which the two‐component system forms a homogeneous mixed phase (assuming a given molar fraction).

In this work, we will discuss the concept of philicity using a relatively simple system consisting of two organic constituents: a regular alkane (lipophilic) and a perfluorinated alkane (fluorophilic), both at a molar fractions x=0.5. Such binary systems have a critical miscibility temperature [13, 14, 15, 16]. Above that temperature, the systems form a single phase; below it, they are phase‐separated. The character of the underlying interactions causing the (im)miscibility of alkanes and perfluoroalkanes is the subject of a long‐standing debate [17, 18, 19, 20]. An early and widespread perception attributes the poor miscibility to unusually weak intermolecular interactions between hydrocarbons and fluorocarbons. This is supported by gas phase measurements of the second virial coefficients [21]. Proposed intrinsic origins of these weak interactions include the size difference between hydrogen and fluorine atoms [21], differences in ionization potential [22, 23], and the low polarizability of fluorine [24]. On the other hand, more recent quantum‐chemical calculations show that alkane‐perfluoroalkane dimer binding energies are stronger, not weaker, than expected from the average of the homo dimer binding energies [25, 26]. Pollice and Chen have instead suggested that the ground‐state geometries of perfluoroalkane molecules are ill‐suited for interacting with alkanes [19]. Another approach by Hasegawa et al. explains the aggregation of perfluoroalkyl chains based on dipole–dipole interactions [27].

Nevertheless, it is still surprisingly difficult to determine the extent to which fundamental atomic properties are responsible for the substantial difference in philicity between alkanes and perfluoroalkanes. In terms of common interaction models, is it the range or depth of the Lennard–Jones‐type van der Waals interaction that causes the difference? We attempt to answer this question by reformulating it in a more quantitative way: How does the mixing/demixing phase transition temperature respond to a change in any of the common Lennard‐Jones parameters? The direct atomistic simulation of such phase transitions is a quite challenging task in itself. We explicitly do not aim to represent either the process or the respective final thermodynamic state as realistically as possible. Such a goal requires extended simulation times and large system sizes. While both these targets are within reach with today's computing resources, achieving convergence in time and size is simply not necessary from our perspective. Instead, we use a more pragmatic approximate description of the mixing process, focusing on the system's *response* to a *change* in philicity of the two components. This response in turn is quantified by the critical miscibility temperature, which itself is computed approximately from pair correlation functions or explicit configurational entropy of mixing.

## 2 Results and Discussion

To analyze how the philicity of individual atoms impacts the miscibility of alkanes and perfluoroalkanes, we conducted over 400 short force field molecular dynamics (MD) simulations of a hexane‐perfluorohexane system. We kept the molar fraction of both constitutents constant at 0.5 and simulated several temperatures between 200 and 300 K. Within the framework of the OPLS‐AA [28] force field, single parameters were systematically varied in several simulation series to artificially induce changes in philicity. We then quantified the system's response based on the compound's miscibility, starting from an initially separated configuration. Further details on the simulation and analysis procedures can be found in the Experimental section.

Preliminary results showed negligible effects of the partial charges in this specific system. This is consistent with dispersion forces being the main source of attraction in alkane‐perfluoroalkane systems [26]. Earlier force fields exist for alkanes [29] and perfluoroalkanes [30] that do not include partial charges at all. Thus, this study focuses on the effects of variations in the energy (ε) and size (σ) parameters of the Lennard‐Jones potential, which represents the van der Waals interactions. All other parameters, including partial charges, were kept at their original OPLS‐AA values [28, 31]. Hereafter, we refer to the van der Waals parameters of a specific atom type ($AT\in \{H,F,C_{H},C_{F}\}$) as ε(AT) and σ(AT), where the subscripts on the carbon atoms indicate the molecule type (hexane or perfluorohexane). This results in a total of eight simulation series for the changes in atomic philicity, as well as a temperature series with unmodified parameters as a reference. Since we are primarily interested in philicity‐induced changes in the mixing behavior, our analyses and discussion are limited to related properties. Macroscopic thermal mechanical properties, such as density and thermal expansion coefficients, were computed and are affected by modifications of the atomic philicities; however, they are not considered here.

### 2.1 Diffusivity

First assessments of miscibility can be made based on molecular mobility. On the length scales used in our simulations, a phase‐separated state usually corresponds to a spherical or slab‐like one‐component phase. This considerably restricts the available diffusion distance as well as the apparent diffusion constant computed from the root mean square distance. For mixed configurations, the mean square displacement can grow comparatively quickly, corresponding to a larger apparent diffusion constant. For our binary system, we computed the self‐diffusion coefficients of hexane and perfluorohexane molecules as a function of the van der Waals parameters. Equation (2) [32, 33] was used to obtain them as the slope of the mean square displacement of the respective molecule, where D denotes the diffusion coefficient, τ the time interval, $r_{i}(t)$ the position of particle i at time t and ⟨…⟩t,i the average over time and all particles.

(2) $$3D=\lim_{\tau \to \infty }\frac{\langle |r_{i}(t+\tau )-r_{i}(t){|}^{2}\rangle t,i}{2\tau }$$

**FIGURE 1:**
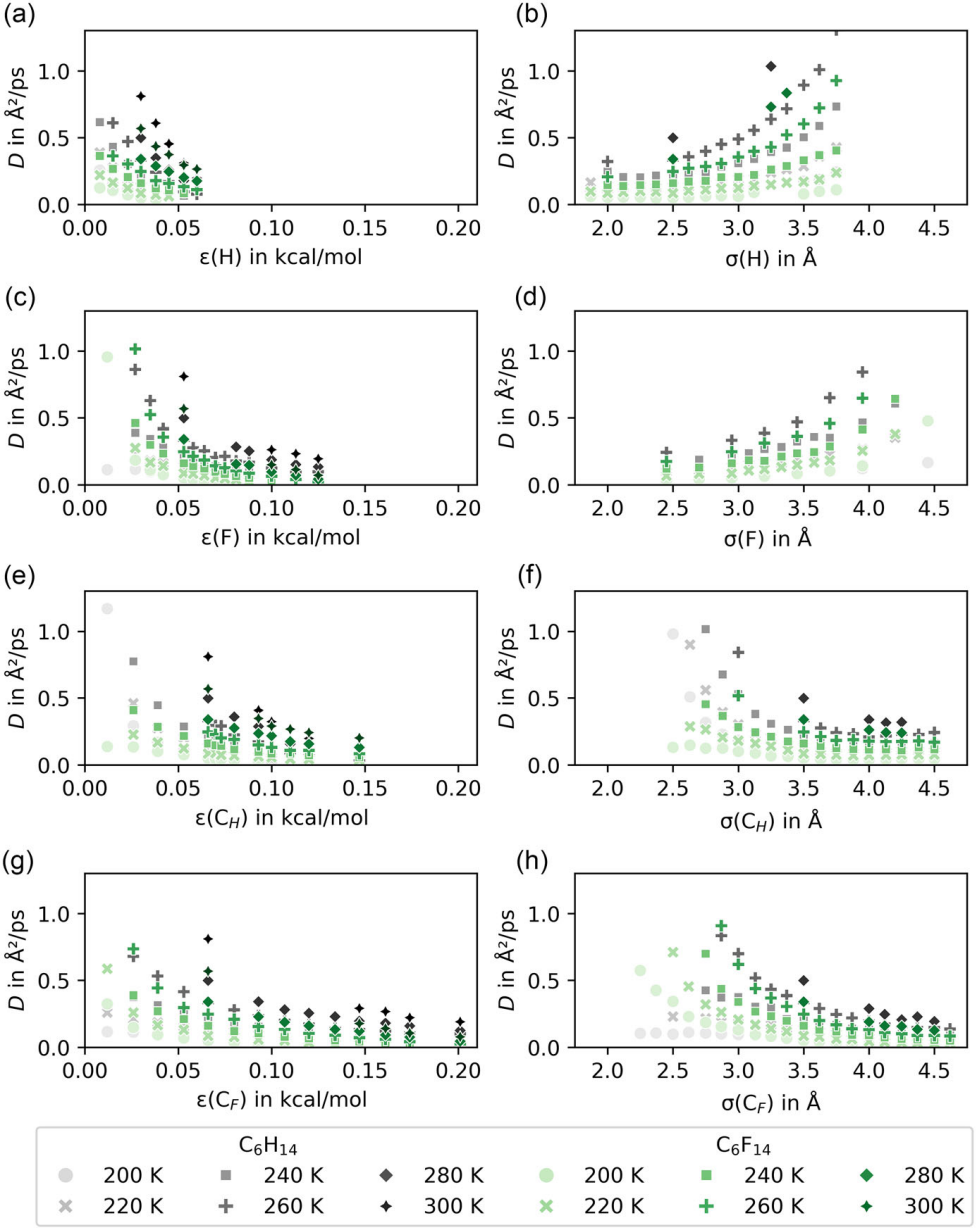
Hexane (gray) and perfluorohexane (green) self‐diffusion coefficients in a 1:1 mixture as a function of the Lennard‐Jones parameters (a) ε(H), (b) σ(H), (c) ε(F), (d) σ(F), (e) *ε*(*C*_H_), (f) *σ*(*C*_H_), (g) *ε*(*C*_F_), and (h) *σ*(*C*_F_). Standard force field (OPLS‐AA) values are ε(H)=0.03 kcal/mol [28], σ(H)=2.5 Å [28], ε(F)=0.053 kcal/mol [31], σ(F)=2.95 Å [31], *ε*(*C*_H_) = 0.066 kcal/mol [28], *σ*(*C*_H_) = 3.5 Å [28], *ε*(*C*_F_) = 0.066 kcal/mol [31], and *σ*(*C*_F_) = 3.5 Å [31].

The characteristic diffusive length scale $\sqrt{6D10\;\mathrm{ns}}$ over the simulation length was compared to the cubic box edge length. All resulting values were on the order of or larger than the simulation box length, indicating that the simulation time is sufficient for diffusion estimates. Figure 1 plots the dependence of the self‐diffusion coefficients on the various energy and size parameters, as well as temperature. In general, smaller hexane molecules diffuse faster than bulkier perfluorohexane molecules. Exceptions to this behavior can be observed when single van der Waals parameters are modified so drastically that the “hexane” or “perfluorohexane” molecules lose their distinct properties. For example, decreasing ε(F) to values below 0.03 kcal/mol, the OPLS value of ε(H) [28], leads to higher perfluorohexane diffusion coefficients than hexane.

At a given temperature, the self‐diffusion coefficients of both types of molecules decrease with increasing ε(H), ε(F), *ε*(*C*_H_), and *ε*(*C*_F_) (see Figure 1a,c,e, and g). The functional form of this decay cannot be represented accurately by fitting to a single exponential or to a simple algebraic function. Similar behavior is also observed in the dependence on *σ*(*C*_H_) and *σ*(*C*_F_) (see Figure 1f,h), while the diffusion coefficients increase with σ(H) and σ(F) (see Figure 1b,d). The underlying reason for the difference in how changing the size parameter of substituent (σ(H), σ(F)) or carbon backbone (*σ*(*C*_H_), *σ*(*C*_F_)) atoms impacts diffusivity remains unclear at this point. Stronger intermolecular interactions are commonly associated with reduced mobility. Thus, the lower self‐diffusion coefficients of hexane and perfluorohexane molecules with larger ε values comply with expectations, as all interactions with the respective H, F, *C*_H_ or *C*_F_ atoms are amplified. The dependency of diffusivity on σ can be rationalized in terms of packing constraints. Within the activated‐jump model, molecular displacement requires the transient creation of a ”hole” (free volume) of suitable size [34, 35, 36]. The probability of a sufficiently large void being formed decreases with higher density, thereby reducing self‐diffusion. For Lennard–Jones fluids, this correlation between density and diffusion coefficients has been reported by various authors [37, 38]. Moreover, Nandi et al. have established a link between the strength of the attractive interactions and the diffusion coefficients as a function of σ in a binary solute‐solvent system [39]. At no or small attractive interaction, diffusivity increases the smaller the solute. In contrast, when strong attractive interactions is applied, the diffusion coefficient first increases until a maximum before decreasing again as σ is decreased [39]. For nonspherical molecules such as hexane and perfluorohexane in an all‐atom force field, this assessment becomes more complex. The different atom types must be considered and displacement becomes orientation‐selective. Our diffusivity data suggests that increasing the size parameter of the outer atoms (σ(H) and σ(F)) affects the intermolecular interactions differently from increasing the size parameters of the carbon atoms (*σ*(*C*_H_) and *σ*(*C*_F_)).

To gain further insight into the driving force of diffusion, the temperature dependence of the self‐diffusion coefficients was analyzed. Diffusion activation energies *E*_A_ and pre‐exponential factors $D_{0}$ were obtained by fitting the data in Arrhenius plots according to Equation (3), where D(T) is the self‐diffusion coefficient at temperature T and R is the gas constant. All plots were confirmed to be linear and screened for outliers. Further information on the fit procedure is provided in Figure S1 and Table S10.

(3) $$\ln\;D(T)=\ln\;D_{0}-\frac{E_{A}}{RT}$$

**FIGURE 2:**
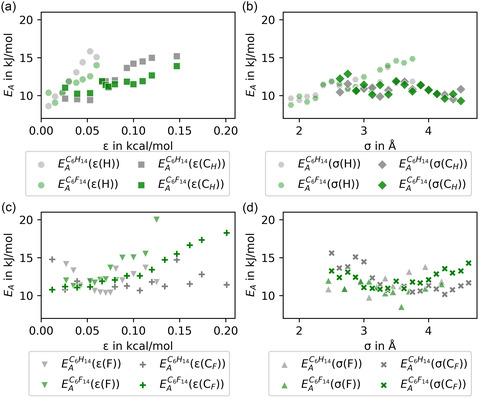
Diffusion activation energies of hexane (gray symbols) and perfluorohexane (green symbols) in a 1:1 mixture as a function of (a) ε(H) and *ε*(*C*_H_), (b) σ(H) and *σ*(*C*_H_), (c) ε(F) and *ε*(*C*_F_), and (d) σ(F) and *σ*(*C*_F_). Standard force field (OPLS‐AA) values are ε(H)=0.03 kcal/mol [28], *ε*(*C*_H_) = 0.066 kcal/mol [28], σ(H)=2.5 Å [28], *σ*(*C*_H_) = 3.5 Å [28], ε(F)=0.053 kcal/mol [31], *ε*(*C*_F_) = 0.066 kcal/mol [31], σ(F)=2.95 Å [31], and *σ*(*C*_F_) = 3.5 Å [31].

The resulting activation energies for the diffusion of hexane and perfluorohexane molecules are compiled in Figure 2, where the individual panels compare how changes in the ε (left) or σ (right) parameters of one type of molecule's atoms influence on the diffusion activation energy of both molecules. Hexane parameters are shown at the top and perfluorohexane parameters at the bottom. In the case of changes in the ε parameters of hexane atoms (see Figure 2a), the diffusion activation energies of both hexane and perfluorohexane are linearly correlated with the parameter value. Changes in ε(H) affect the diffusion activation energies roughly twice as much as changes in *ε*(*C*_H_). Furthermore, the diffusion activation energy of hexane rises at a slightly faster rate than that of perfluorohexane with changes in both ε(H) and *ε*(*C*_H_). Similar effects are observed for the diffusion activation energy as a function of ε(F) and *ε*(*C*_F_) in Figure 2c. Here, the distinction between the types of molecules becomes clearer. The perfluorohexane diffusion activation energy is strongly linearly correlated with both ε(F) and *ε*(*C*_F_), while the impact of changes in them on the hexane diffusion activation energy is close to zero. The absolute impact of changes in ε(F) is approximately twice that of *ε*(*C*_F_). In the absolute‐rate/transition‐state picture of diffusion in liquids, self‐diffusion proceeds via thermally activated transitions between locally stable packing configurations (”normal states”) [35, 40, 41]. Thus, the extracted diffusion activation energies can be interpreted as effective barriers controlling the rate of such elementary rearrangement events. The linear correlations between *E*_A_ and ε observed in Figure 2 are therefore consistent with shifts of the effective activation barriers for molecular rearrangement as the depth of the pairwise interaction potentials (ε) is varied. In contrast, changes in the σ values generally have a smaller impact on the diffusion activation energy. Changes in only one of the σ values, σ(H), indicate a correlation with the diffusion activation energy. This correlation is surprisingly more pronounced for the diffusion of perfluorohexane than hexane (compare Figure 2b). Changes in *σ*(*C*_H_), σ(F), and *σ*(*C*_F_) lead to diffusion activation energies that fluctuate in the range of 8–15 kJ/mol, with no clear dependence on σ (see Figure 2b,d).

Overall, the relationship between the strength of the van der Waals forces and diffusion is complex, but can be interpreted in terms of activated transport in a crowded liquid environment. The magnitude of ε directly influences diffusion activation energies. Increasing the depth of the potential well results in stronger interactions with atoms of the respective type. Since this affects the motion of both hexane and perfluorohexane molecules, the kinetics of their mixing process is influenced by changes in ε. The strength of this influence varies with the changed parameter. The greatest impact was found when changing the values of ε(H) on hexane diffusion barriers and the values of ε(F) on perfluorohexane diffusion barriers. However, changing the ε values of hexane atoms seems to have a stronger effect on diffusion activation energies than changing the ε values of perfluorohexane atoms. This can be understood through a packing argument. Due to its smaller molecular size, hexane can approach neighboring molecules more closely and form stronger local contacts. Consequently, increasing ε more efficiently strengthens local cohesion and increases the effective rearrangement barrier than an analogous change in ε for the bulkier perfluorohexane.

Second, the lack of correlation between the activation energies and σ, despite the clear dependence of the diffusion coefficients on these parameters, indicates that the influence of σ on diffusivity must be entropic rather than enthalpic. Consequently, the pre‐exponential factor should be dependent on changes in σ values and not depend on changes in ε values. For the latter, we observed a dependence of the enthalpic barriers. A complementary plot of the pre‐exponential factors, $D_{0}$, as a function of the different interaction parameters is provided in Figure S2. Indeed, no correlation between $D_{0}$ and the parameters ε(H), *ε*(*C*_H_), ε(F), and *ε*(*C*_F_) is observed. In contrast, there is a clear connection between the pre‐exponential factor and the value of σ. $D_{0}$ increases exponentially with σ(H) and σ(F), while it decreases exponentially with the size of the carbon atoms *σ*(*C*_H_) and $\sigma (C_{F})$.

Thus, regarding the Arrhenius equation (Equation (3)), we conclude that the explicit dependencies of the fundamental constituents ($D_{0}$, *E*_A_) on the van der Waals parameters (ε, σ) can be reformulated as follows:

(4) $$\ln\;D(\sigma_{i},\varepsilon_{i},T)=\ln\;D_{0}(\sigma_{i})-\frac{E_{A}(\varepsilon_{i})}{RT}$$

### 2.2 Temperature‐Dependent Mixing Behavior

**FIGURE 3:**
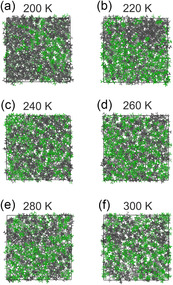
Trajectory snapshots after 10 ns simulation of a hexane‐perfluorohexane system with OPLS‐AA parameters [28, 31, 42] at different temperatures. (a) 200 K, (b) 220 K, (c) 240 K, (d) 260 K, (e) 280 K, and (f) 300 K (reprinted from Upterworth and Sebastiani, used under CC‐BY 4.0 license [11]). For easier distinction, hexane molecules are colored in gray, and perfluorohexane molecules in green.

The primary objective of this study is to quantify the impact of the individual Lennard–Jones potential parameters on the temperature‐dependent mixing behavior of alkane‐perfluoroalkane mixtures. Particular emphasis is placed on the critical solution temperature, i.e., the temperature at which the phase transition from separate to mixed phases occurs. A multitude of methodologies exist for deriving the temperature of miscibility from molecular dynamics trajectories. The first and most elementary method is visual inspection. Figure 3 exemplary shows representative snapshots of the temperature series of simulations with unmodified OPLS‐AA parameters [28, 31, 42]. At temperatures as low as 200 and 220 K, hexane and perfluorohexane remain mostly separated. As the temperature rises, the two compounds gradually mix more from 240 to 300 K. However, given the spatial heterogeneity of the densities of the two molecules and periodic boundary conditions, it is challenging to characterize the state of mixing more precisely with the human eye.

Overall, the miscibility temperatures in our simulations are substantially lower than the experimentally observed 296 K upper critical solution temperature (UCST) of the hexane‐perfluorohexane system [15, 16]. Similar findings have been reported by other authors. Song et al. found that standard force fields and combining rules do not accurately reflect the tendency toward segregation in binary alkane‐perfluoroalkane mixtures [18]. This was confirmed by Zhang and Siepman, who found that the UCST was underestimated by more than 30% when using the Lorentz–Berthelot combining rules [43]. In other cases, alkane‐perfluoroalkane immiscibility was overestimated. Different theoretical studies overpredicted the UCST of such systems using soft associating fluid theory (SAFT) approaches [44, 45]. Adding to the complexity, Morgado et al. observed nano‐segregation of hexane and perfluorohexane above the UCST using 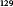
Xe nuclear magnetic resonance spectroscopy and molecular dynamics simulations [42]. These results demonstrate the difficulty of accurately describing the liquid–liquid equilibria of alkane‐perfluoroalkane systems. However, as stated in the introduction, that is not the objective of this study. Instead, we are interested in the impact of changes in the van der Waals potential parameters. Since we are comparing these relative to each other, deviations from the experimental miscibility temperature are not a major concern.

**FIGURE 4:**
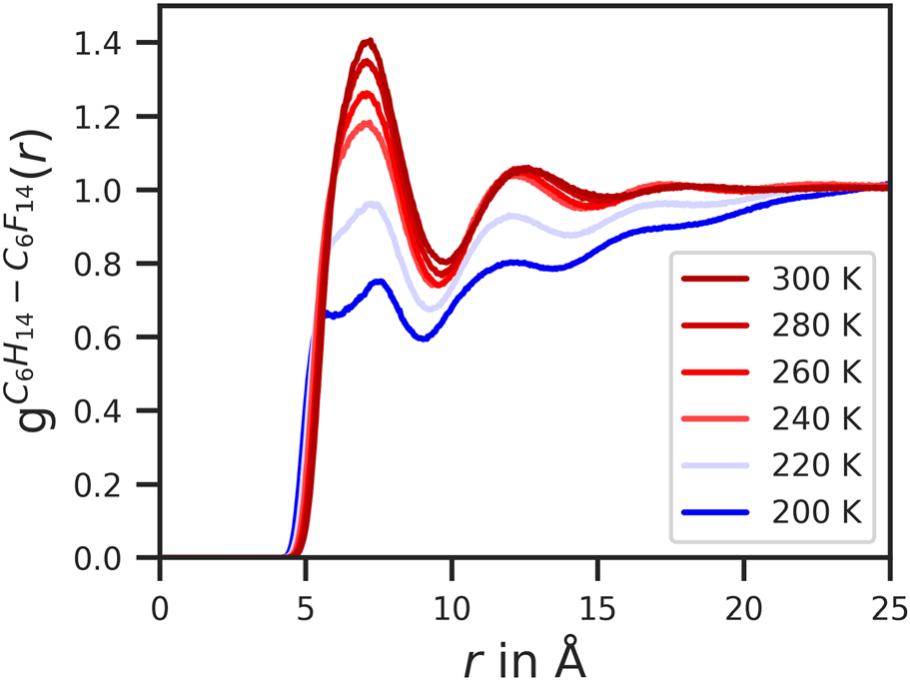
Temperature dependence of the pair correlation function $g^{C_{6}H_{14}-C_{6}F_{14}}(r)$ for simulations of a 1:1 hexane–perfluorohexane mixture with unmodified OPLS‐AA parameters.

A more quantitative approach to obtaining the temperature of miscibility is offered by pair correlation functions, whose peak height can be used to differentiate between mixed and separated states [11, 46, 47]. Figure 4 shows the pair correlation functions, $g^{C_{6}H_{14}-C_{6}F_{14}}(r)$, between the centers of mass of hexane and perfluorohexane molecules at different simulation temperatures. The shape of the pair correlation functions remains unchanged within the simulated temperature range, exhibiting two maxima at distances of ≈7.5 and 12 Å. At larger distances, g(r) approaches uniform density. However, our focus remains on the first maximum, which represents the nearest neighbors of perfluorohexane molecules. The greater the peak height, the greater the degree of mixing. A peak height below uniform density indicates phase separation. In our simulation series with unmodified OPLS parameters, the height of the first maximum increases with temperature and crosses the uniform density threshold between 220 and 240 K. This confirms our previous observation that hexane and perfluorohexane remain in separate phases at 200 and 220 K, while mixing increasingly at higher temperatures.

**FIGURE 5:**
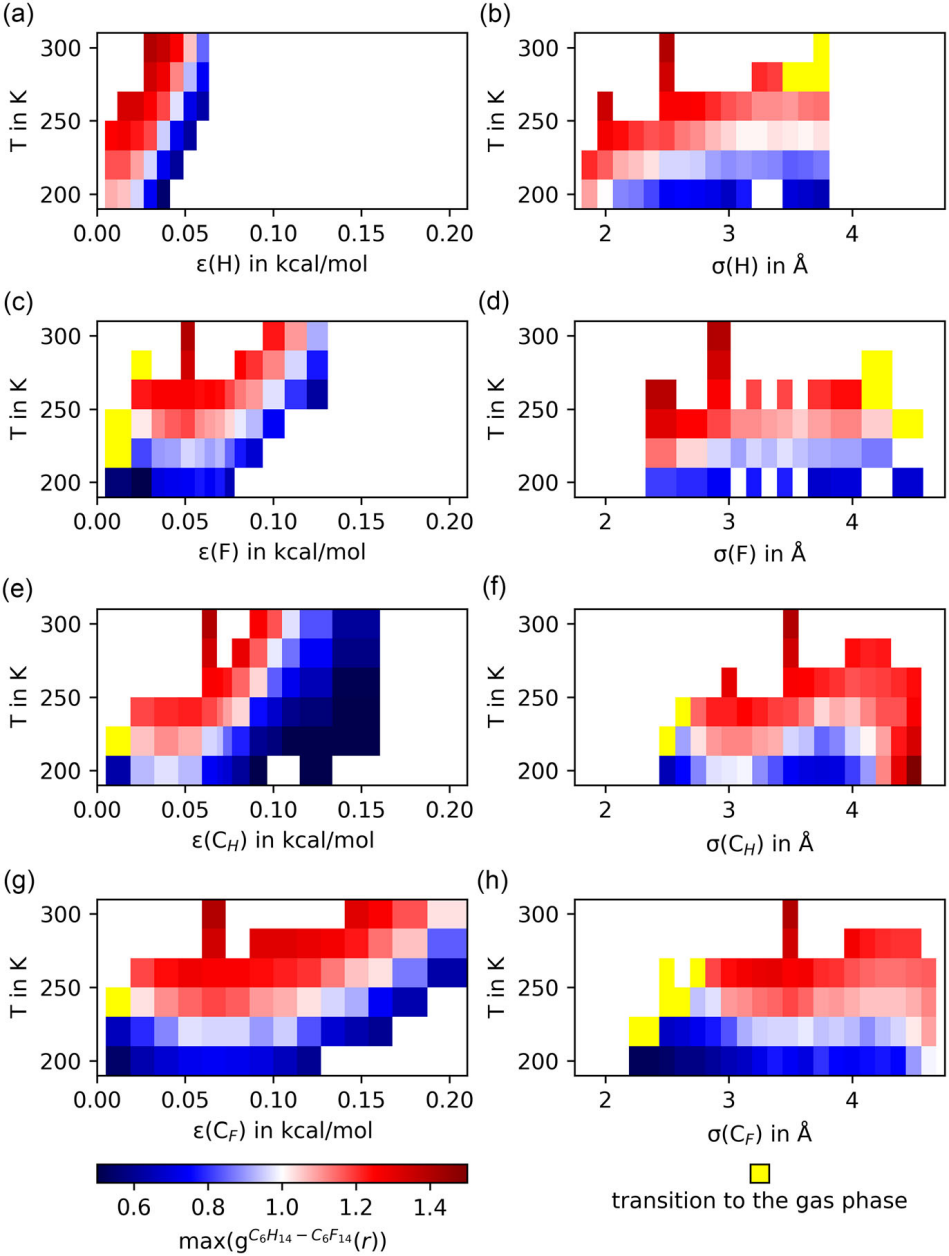
Temperature and Lennard–Jones parameter dependent state of mixing of the hexane‐perfluorohexane system. Separated (blue) and mixed (red) configurations are distinguished by the height of the first peak of the pair correlation function between hexane and perfluorohexane molecules, $\max(g^{C_{6}H_{14}-C_{6}F_{14}}(r))$. Standard force field (OPLS‐AA) values are ε(H)=0.03 kcal/mol [28], σ(H)=2.5 Å [28], ε(F)=0.053 kcal/mol [31], σ(F)=2.95 Å [31], *ε*(*C*_H_) = 0.066 kcal/mol [28], *σ*(*C*_H_) = 3.5 Å [28], *ε*(*C*_F_) = 0.066 kcal/mol [31], and *σ*(*C*_F_) = 3.5 Å [31].

At this point, we would like to introduce a color code based on the height of the first peak of $g^{C_{6}H_{14}-C_{6}F_{14}}(r)$ that reflects the state of mixing. Blue colors are used for separated phases ($\max(g^{C_{6}H_{14}-C_{6}F_{14}}(r))<1.0$) and red for mixed phases ($\max(g^{C_{6}H_{14}-C_{6}F_{14}}(r))>1.0$). The larger the gap to the uniform density, the darker the color. This color code is also used in Figure 5, where the state of mixing is plotted as a function of temperature and parameter value for each varied Lennard–Jones parameter. The few yellow boxes represent simulations that yielded a transition to gas phase.

A semi‐quantitative estimate of the miscibility temperature for a given set of parameters can be obtained by identifying the temperature at which the system's configuration transitions from a separated (blue) to a mixed state (red). For all ε, we observe a positive correlation between the miscibility temperature and the magnitude of ε over the majority of the parameter range. The shift in the miscibility temperature is most pronounced with an increase in ε(H), followed by *ε*(*C*_H_) and ε(F). Increasing *ε*(*C*_F_) has the least impact (compare Figure 5a,c,e,g). Changing the ε of hexane atoms has a greater effect on the miscibility of alkanes and perfluoroalkanes than changing the ε of perfluorohexane atoms. This is particularly noticeable for variations in *ε*(*C*_H_) and *ε*(*C*_F_), which originally have the same OPLS‐value of 0.066 kcal/mol [28, 31]. The underlying reason for this effect is the difference in size between the molecules. Smaller hexane molecules have more and closer contacts. Thus, changes in hexane‐related parameter values strongly enhance hexane–hexane interactions and favor phase separation. It is also noteworthy that, for very small values of ε(F), *ε*(*C*_F_) and *ε*(*C*_H_) below 0.05 kcal/mol, the trend in the miscibility temperature reverses. While there is no simple qualitative explanation for this observation, a recently published tool to analyze the effective interaction strength [11] could provide further insights.

Unlike ε, the temperature of miscibility is insensitive to changes in σ. While there is fluctuation in the peak height of $g^{C_{6}H_{14}-C_{6}F_{14}}(r)$, most miscibility temperatures fall within the range of 220 to 240 K. Although short run times could contribute to these fluctuations, our diffusion analysis and systematic shifts in miscibility behavior when varying ε indicate that the small variations in the observed miscibility are inherent to the value of σ. This is supported by discernible patterns within the data despite the small overall effect of changing σ. As with diffusion coefficients, changes in the size of the outer atoms (H and F) have a different effect than changes in the size of the carbon atoms. For σ(H) and σ(F), the miscibility temperature slowly increases with the parameter values before it decreasing slightly at very large values (σ(H)>3.5 Å and σ(F)>4.2 Å). Conversely, for changes in *σ*(*C*_H_) and *σ*(*C*_F_), we observe several maxima in the miscibility temperature at *σ*(*C*_H_) = 2.63  Å, *σ*(*C*_H_) = 3.75 Å, *σ*(*C*_F_) = 2.75 Å and *σ*(*C*_F_) = 4.13 Å. Interestingly, transition into the gas phase is observed for very large values of σ(H) and σ(F), but for very small values of *σ*(*C*_H_) and *σ*(*C*_F_). This difference can be understood with a simple qualitative picture. Increasing the size of the outer substituent atoms (H and F) leads to larger particle distances and ultimately to transitioning to the gas phase. One would expect the same behavior to occur for very large values of *σ*(*C*_H_) and *σ*(*C*_F_), outside the simulated range. Reducing the size of the carbon atoms, on the other hand, forces the molecules into close contact. The strong repulsive forces induced in this way lead to a transition to the gas phase. A more quantitative analysis of the strength of the underlying individual pairwise interactions would be highly informative but would exceed the scope of this study. We will address this, as well as the behavior for small values of ε, in a subsequent study.

The comparatively minor influence of changes in σ on the miscibility is consistent with the general understanding. Song et al. found that different combining rules that aim to more accurately describe the properties of alkane‐perfluoroalkane mixtures do not include changes in σ, focusing instead on ε [18]. A common strategy is to reduce the strength of the unlike intermolecular interactions relative to the geometric mean [18, 42, 43, 45]. By adapting ε, the enthalpic contribution changes directly and affects miscibility. As discussed in the section on diffusivity, changes in σ appear to have an entropic influence.

### 2.3 Entropy of Mixing

Although extracting the miscibility temperature from the height of the peaks in the pair correlation function yields local, structure‐based results, the process involves several steps and is not easily comprehensible. Therefore, we looked for a simpler descriptor that would directly quantify the state of mixing from a simulation trajectory. A recently published approach from our group for calculating configurational entropy of mixing [9] offers exactly that. Here, we are testing its applicability in determining miscibility temperatures from temperature series of molecular dynamics simulations.

**FIGURE 6:**
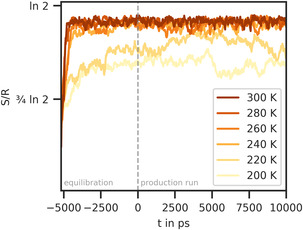
Entropy of mixing (in units of R) of hexane and perfluorohexane during simulations with OPLS force field parameters at different temperatures.

Figure 6 shows the temporal evolution of the configurational entropy of mixing for the simulation series with unmodified OPLS parameters for hexane [28] and perfluorohexane [31] at different temperatures. Starting from a configuration in which hexane and perfluorohexane are in two separate layers, the configurational entropy of mixing quickly increases during equilibration as the molecules begin to move and mix. Then, it converges to a value representing the mixed state. This converged value is very similar for simulations at temperatures above 240 K. At the two low temperatures of 200 and 220 K, the entropy of mixing fluctuates more, but the overall value is lower, which corresponds to partially mixed configurations. Even at higher temperatures, the attained values for the fully mixed states do not reach the theoretical value of ln 2 for equimolar mixtures. The same applies to the configurational entropy at the start of the simulation, which is substantially greater than the ideal value for completely separated phases. These deviations are a consequence of the small size of the simulated system, in which our initial arrangement of two separate layers corresponds to a partially mixed state of the large periodic system. It has recently been shown that the configurational entropy converges to theoretical limits as the number of molecules increases, though very large systems would be necessary to reach these limits [48]. Nevertheless, further insights can be gained from the evolution of the entropy‐time curves.

To obtain a more accurate estimate of the temperature of miscibility, we performed additional simulations at temperatures between the existing ones. We then computed the configurational entropy of mixing throughout the whole equilibration and production runs. Finally, we fitted the resulting entropy curves with an exponential function (Equation (5)). Here, $S_{0}+S_{\infty }$ is the converged entropy value, $S_{\mathrm{conv}}(T)$, at the respective temperature.

(5) $$S(t)=S_{0}+S_{\infty }\left(1-\exp\;\left(-\frac{t}{\tau }\right)\right)$$

**FIGURE 7:**
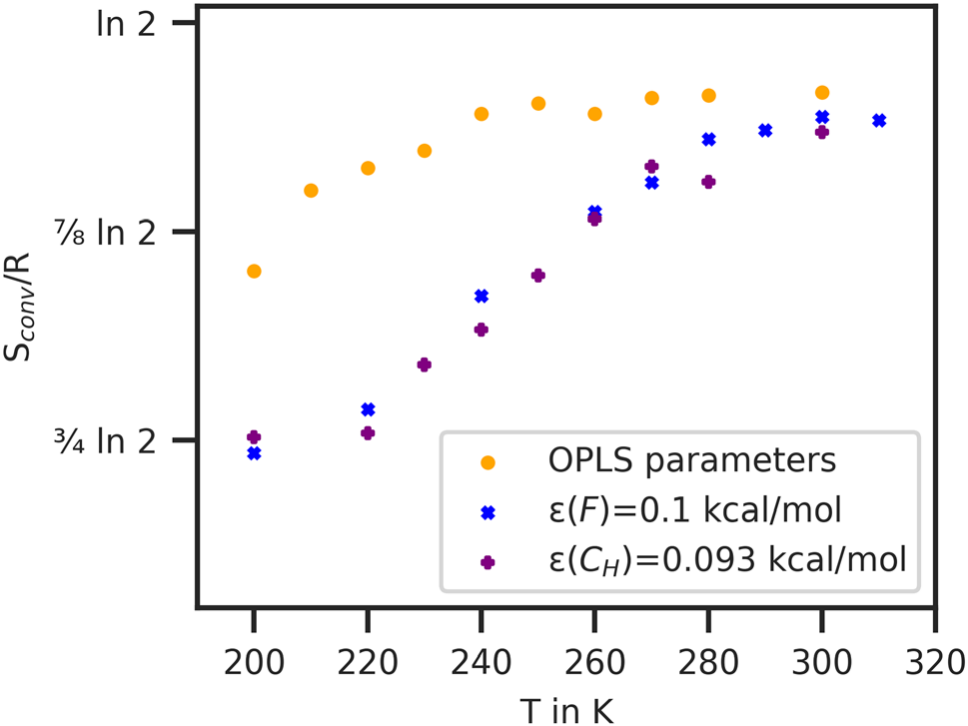
Entropy of mixing (in units of R) as a function of temperature for three different parameter settings. Orange dots, unmodified OPLS parameters (ε(F)=0.053 kcal/mol [31], *ε*(*C*_H_) = 0.066 kcal/mol [28]); blue crosses, ε(F) increased by 90% (=0.1 kcal/mol); purple plus signs, *ε*(*C*_H_) increased by 40% (=0.093 kcal/mol).

The temperature dependence of these converged entropies is compared in Figure 7 for unmodified OPLS parameters and two series with modified energy parameters. Specifically, we consider an increase of ε(F) by about 90% from 0.053 to 0.1 kcal/mol and an increase of *ε*(*C*_F_) by about 40% from 0.066 to 0.093 kcal/mol. The similarity of the $S_{\mathrm{conv}}(T)$ curves for these two alterations is purely fortuitous. They are S‐shaped, exhibiting a lower degree of mixing at low temperatures and a steep increase before reaching the plateau of complete mixing at higher temperatures. Analogous to other first‐order phase transitions, the inflection point of the entropy curve can be interpreted as the critical temperature of miscibility. For the unmodified OPLS parameters, the inflection point occurs at such low temperature that the S‐shape of the curve is barely discernible. The miscibility temperature between 200 and 220 K is slightly lower than that determined by visual inspection and pair correlation functions. Lower miscibility temperatures than those obtained from pair correlation function analysis are also obtained for the modified energy parameters. Specifically, the entropy inflection lies between between 240 and 260 K for ε(F)=0.1 kcal/mol and between 240 and 250 K for *ε*(*C*_H_) = 0.093 kcal/mol. In contrast, the peak height of the pair correlation functions indicates miscibility temperatures of 260–280, and 240–260 K respectively. The transition from separate to mixed configurations does not occur instantly, but rather spans a wide temperature range of around 40 K, which is clearly too large in comparison to experiment. This discrepancy results from the choice of simulation parameters, which is further explained in Section 3.3 of the Supporting Information. One could also alternatively define the miscibility temperature as the temperature, at which the system is fully mixed and the entropy of mixing reaches its plateau. This yields miscibility temperatures of ≈ 250 K for unmodified OPLS parameters, 290 K for ε(F)=0.1 kcal/mol, and 280 K for *ε*(*C*_H_) = 0.093 kcal/mol. These temperatures are higher than those from the pair correlation functions but closer to the experimental upper critical solution temperature. Regardless of the exact definition of the miscibility temperature, it is clear that increasing the energy parameter of the van der Waals interaction has an influence. Increasing both ε(F) from 0.053 to 0.1 kcal/mol and *ε*(*C*_H_) from 0.066 to 0.093 kcal/mol shifts the miscibility temperature to higher temperatures of 30–40 K. These two changes coinciding is purely accidental. However, this demonstrates once again that changes to the energy parameters of smaller hexane molecules have a greater impact on miscibility than changes to those of perfluorohexane molecules. Increasing *ε*(*C*_H_) by 40% has the same effect as increasing ε(F) by 90%.

Thus, we have demonstrated that the configurational entropy of mixing approach [9] is suitable for obtaining and comparing miscibility temperatures from molecular dynamics simulations. The state of mixing can be accessed instantly from the simulation trajectory without averaging over many steps. Short simulation lengths and fit windows suffice to estimate the system's tendency toward mixture or segregation.

## 3 Conclusion

In summary, we conducted a systematic study of the fundamental origins of the miscibility and immiscibility in binary molecular liquids. Specifically, we studied this on the example of alkanes and perfluoroalkanes. We quantify the degree of mixing as the system's response to changes in atomic philicity via two descriptors: one based on the pair correlation function and one based on a recently developed state function that measures instantaneous configurational entropy of mixing. As the degree of mixing varies with temperature, we compute a critical temperature of miscibility derived from the descriptors.

Our focus is on the relationship between the miscibility temperature and the microscopic interaction parameters of the two constituents, here specifically the parameters that describe the van der Waals interactions via Lennard–Jones functions in the atomistic force field. We observe that the miscibility temperature is strongly correlated with all interatomic interaction energy parameters ε, but almost uncorrelated with parameters related to atomic radii σ. Interestingly, the impact of the carbon van der Waals parameter depends on the type of carbon, i.e., whether it is a CH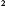 or a CF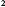 carbon. Interaction energy parameters related to smaller hexane molecules have a greater influence miscibility more than parameters related to larger perfluorohexane molecules.

An interesting side result was obtained for the diffusion constants of the binary mixtures. We decomposed diffusion into a temperature‐independent prefactor and a temperature‐dependent part described by the Arrhenius equation. We observe that the Arrhenius activation energy is practically linearly correlated with the atomic interaction energy parameters. However, the temperature‐independent pre‐exponential part of the diffusion constant shows the exact opposite behavior. It is strongly correlated with the atomic size parameters but uncorrelated to the atomic interaction energy parameters.

## 4 Experimental

Molecular dynamics simulations utilizing the OPLS‐AA [28] force field were used to study how changes in the philicity of alkanes and perfluoroalkanes affect their mixing behavior. The philicity of individual atoms was expressed by the energy and size parameters of the Lennard‐Jones representation of van der Waals forces. These parameters were systematically varied in eight simulation series of a hexane–perfluorohexane mixture. In each series, one parameter (ε(H), ε(F), *ε*(*C*_H_), *ε*(*C*_F_), σ(H), σ(H), σ(F), *σ*(*C*_H_) or *σ*(*C*_F_)) was varied while the others were kept at their original OPLS‐AA values for alkanes [28] and perfluorinated alkanes [31]. Table 1 provides an overview of the considered parameter ranges. Full lists of all performed simulations are provided in Tables S1–S9. As is common within the OPLS framework, the geometric mean rule was used for ε and σ of dissimilar pairwise interactions. An exception is the H–F interaction, for which special mixing rules suggested by Morgado et al. [42] were applied according to Equation (6).

**TABLE 1:** Lennard‐Jones parameter values used in molecular dynamics simulations of the $C_{6}H_{14}-C_{6}F_{14}$ mixture. Energy parameters, ε, are given in kcal/mol and size parameters, σ, in Å.

Parameter	OPLS value	Range of simulated values
ε(H)	0.03 [28]	0.008–0.060
ε(F)	0.053 [31]	0.012–0.125
*ε*(*C*_H_)	0.066 [28]	0.012–0.147
*ε*(*C*_F_)	0.066 [31]	0.012–0.201
σ(H)	2.5 [28]	1.87–3.75
σ(F)	2.95 [31]	2.45–4.45
*σ*(*C*_H_)	3.5 [28]	2.50–4.50
*σ*(*C*_F_)	3.5 [31]	2.25–4.62

(6) $$\varepsilon_{\mathrm{HF}}=0.8\sqrt{\varepsilon_{H}\varepsilon_{F}}\;\sigma_{\mathrm{HF}}=1.04\sqrt{\sigma_{H}\sigma_{F}}$$

To avoid the long simulation times associated with phase separation processes, we observed the (partial) mixing process starting from an initially separated configuration. A cubic box containing two layers of 250 hexane and 250 perfluorohexane molecules was built using PACKMOL [49]. This box was used as the initial configuration for all molecular dynamics simulations, which were carried out using the LAMMPS [50, 51] program package. Prior to the production runs, energy minimization and multi‐step equilibration, including box resizing, were performed in the same manner as previously described [11]. Production runs were run for 10 ns with a time step of 1 fs in the NVT ensemble. Temperature control was achieved using Nosé‐Hoover thermostats [52, 53, 54] with a coupling constant of 100 fs. Pairwise non‐bonded interactions were considered up to a cutoff distance of 8 Å combined with the PPPM (particle–particle particle‐mesh) solver [55] for long‐range Coulomb interactions.

Trajectory snapshots were rendered from VMD [56] with Tachyon [57], and further trajectory analysis was carried out using TRAVIS [32, 33]. The mean squared displacements and pair correlation functions were averaged over the entire of the production run. Data points were taken every 1000 fs for the series with modified *σ*(*C*_H_) and *σ*(*C*_F_) and every 100 fs for all other simulations. Self‐diffusion coefficients were obtained from the mean squared displacement of hexane and perfluorohexane molecules (Equation (2)). The pair correlation functions $g^{C_{6}H_{14}-C_{6}F_{14}}(r)$ were computed between the centers of mass of the molecules. The numerical values of all obtained $D(C_{6}H_{14})$, $D(C_{6}F_{14})$, further details on the Arrhenius analysis, and the heights of the first peak of the pair correlation functions ($\max g^{C_{6}H_{14}-C_{6}F_{14}}(r)$) are provided in Tables S1–S10 and Figure S1.

The configurational entropy of mixing was calculated every 200 fs during the equilibration and production runs by using our EntMix software package [9]. An identical smearing parameter of 3.45 Å was used for broadening the particle densities with Gaussian functions. This particular value is the mean of the optimal smearing parameters for pure hexane and perfluorohexane [48]. Subsequent exponential fits of the entropy curves (Equation (5)) were performed for data points over the entire equilibration and the first 4.5 ns of the production runs. Entropy‐time curves and exponential fit coefficients are reported in Figures S3–S5 and Table S11. Finally, all plots were created using the Python libraries Matplotlib [58] and Seaborn [59].

## Supporting Information

Additional supporting information can be found online in the Supporting Information section.

## Funding

This work was supported by the Deutsche Forschungsgemeinschaft (436494874).

## Conflicts of Interest

The authors declare no conflicts of interest.

## Supporting information

Supplementary Material

## Data Availability

The data that support the findings of this study are available from the corresponding author upon reasonable request.
