# Supplementary material for: Molecular Origins of Philicity: How Atomic Interactions Determine Miscibility and Diffusivity
Source: Chemphyschem. 2026 Apr 25;27(8):e202500875. doi: 10.1002/cphc.202500875 (PMC13110110; doi:10.1002/cphc.202500875)
Supplement: Supplementary file 1 — Supplementary Material [file CPHC-27-e202500875-s001.pdf]

Supporting Information

Molecular Origins of Philicity: How atomic interactions determine miscibility and diffusivity

Anna Luisa Upterworth, Tom Erik Steinkopf, Daniel Sebastiani

Institute of Chemistry, Martin Luther University Halle-Wittenberg, 06120 Halle, Germany

Contents

1 Molecular dynamics simulations **S2**
1.1 Simulations with standard force field parameters . . . S2
1.2 Simulations with modified Lennard-Jones energy parameters . . . S3
1.3 Simulations with modified Lennard-Jones size parameters . . . S10

2 Temperature dependence of self-diffusion coefficients **S19**
2.1 Arrhenius type analysis . . . S19
2.2 Pre-exponential factors of diffusion . . . S23

3 Configurational entropy of mixing **S24**
3.1 Additional entropy-time curves . . . S24
3.2 Exponential fitting of the entropy-time curves . . . S26
3.3 Entropy of mixing as a function of system size . . . S27

1 Molecular dynamics simulations

1.1 Simulations with standard force field parameters

Table S1: List of molecular dynamics simulations performed on a C_6H_{14}-C_6F_{14} mixture (molar fraction $x = 0.5$) using the OPLS-AA[1, 2, 3] force field parameters. In addition, the obtained self-diffusion coefficients, the characteristic diffusive length scales and the height of the first peak in the pair correlation function between the centers of mass of the hexane and perfluorohexane molecules are given.

T in K	edge length in Å	ρ in g/cm^3	$D^{C_6H_{14}}$ in Å^2/ps	$\sqrt{6D^{C_6H_{14}}10\,\mathrm{ns}}$ in Å	$D^{C_6F_{14}}$ in Å^2/ps	$\sqrt{6D^{C_6F_{14}}10\,\mathrm{ns}}$ in Å	max $\left(g^{C_6H_{14}-C_6F_{14}}(r)\right)$
200	50.56	1.36	0.071	65.27	0.051	55.32	0.74
220	51.33	1.30	0.135	90.00	0.085	71.41	0.96
240	52.26	1.23	0.208	111.71	0.161	98.29	1.18
260	53.32	1.16	0.334	141.56	0.248	121.98	1.26
280	54.6	1.08	0.498	172.86	0.341	143.04	1.34
300	56.75	0.96	0.81	220.45	0.568	184.61	1.4
210*	50.92	1.33	–	–	–	–	–
230*	51.8	1.27	–	–	–	–	–
250*	52.78	1.2	–	–	–	–	–
270*	53.98	1.12	–	–	–	–	–

*Additional simulation for configurational entropy of mixing calculation

1.2 Simulations with modified Lennard-Jones energy parameters

Variation of $\varepsilon(H)$

Table S2: List of molecular dynamics simulations performed on a C_6H_{14}-C_6F_{14} mixture (molar fraction $x = 0.5$) with varied energy parameter $\varepsilon(H)$. The obtained self-diffusion coefficients, the characteristic diffusive length scales and the height of the first peak in the pair correlation function between the centers of mass of the hexane and perfluorohexane molecules are also provided.

$\varepsilon(H)$ in kcal/mol	T in K	edge length in Å	ρ in g/cm^3	$D^{C_6H_{14}}$ in Å^2/ps	$\sqrt{6D^{C_6H_{14}}10\,\text{ns}}$ in Å	$D^{C_6F_{14}}$ in Å^2/ps	$\sqrt{6D^{C_6F_{14}}10\,\text{ns}}$ in Å	max $\left(g^{C_6H_{14}-C_6F_{14}}(r)\right)$
0.008	200	51.93	1.26	0.258	124.42	0.125	86.8	1.08
0.008	220	53.18	1.17	0.39	152.97	0.221	115.15	1.17
0.008	240	54.95	1.06	0.615	192.09	0.365	147.99	1.25
0.015	200	51.38	1.3	0.155	96.44	0.106	79.75	1.06
0.015	220	52.36	1.23	0.276	128.69	0.165	99.50	1.17
0.015	240	53.65	1.14	0.43	160.62	0.268	126.81	1.27
0.015	260	55.22	1.05	0.61	191.31	0.365	147.99	1.33
0.023	200	50.85	1.34	0.111	81.61	0.073	66.18	0.96
0.023	220	51.78	1.27	0.188	106.21	0.123	85.91	1.09
0.023	240	52.77	1.2	0.305	135.28	0.206	111.18	1.23
0.023	260	54.05	1.11	0.471	168.11	0.304	135.06	1.33
0.038	200	50.27	1.39	0.044	51.38	0.04	48.99	0.55
0.038	220	51	1.33	0.073	66.18	0.074	66.63	0.73
0.038	240	51.78	1.27	0.144	92.95	0.121	85.21	0.95
0.038	260	52.72	1.2	0.242	120.50	0.179	103.63	1.18
0.038	280	53.9	1.12	0.35	144.91	0.289	131.68	1.28
0.038	300	55.38	1.04	0.608	191.00	0.436	161.74	1.36
0.045	220	50.71	1.35	0.053	56.39	0.062	60.99	0.61
0.045	240	51.51	1.29	0.1	77.46	0.107	80.12	0.73
0.045	260	52.33	1.23	0.172	101.59	0.159	97.67	0.97
0.045	280	53.33	1.16	0.26	124.90	0.247	121.74	1.12
0.045	300	54.61	1.08	0.454	165.05	0.375	150.00	1.26
0.053	240	51.22	1.31	0.065	62.45	0.084	70.99	0.62
0.053	260	52.01	1.25	0.096	75.89	0.134	89.67	0.72
0.053	280	52.92	1.19	0.18	103.92	0.203	110.36	0.93
0.053	300	53.97	1.12	0.312	136.82	0.296	133.27	1.06
0.06	260	51.8	1.27	0.078	68.41	0.112	81.89	0.63
0.06	280	52.61	1.21	0.113	82.34	0.177	103.05	0.74
0.06	300	53.64	1.14	0.199	109.27	0.266	126.33	0.85

Variation of $\varepsilon(F)$

Table S3: List of molecular dynamics simulations performed on a C_6H_{14}-C_6F_{14} mixture (molar fraction $x = 0.5$) with varied energy parameter $\varepsilon(F)$. The obtained self-diffusion coefficients, the characteristic diffusive length scales and the height of the first peak in the pair correlation function between the centers of mass of the hexane and perfluorohexane molecules are also provided.

$\varepsilon(F)$ in kcal/mol	T in K	edge length in Å	ρ in g/cm^3	$D^{C_6H_{14}}$ in Å^2/ps	$\sqrt{6D^{C_6H_{14}}10\,\text{ns}}$ in Å	$D^{C_6F_{14}}$ in Å^2/ps	$\sqrt{6D^{C_6F_{14}}10\,\text{ns}}$ in Å	$\max\left(g^{C_6H_{14}-C_6F_{14}}(r)\right)$
0.012	200	59.89	0.82	0.113	82.34	0.956	239.50	0.56
0.012	220†	–	–	–	–	–	–	–
0.012	240†	–	–	–	–	–	–	–
0.027	200	52.21	1.24	0.115	83.07	0.184	105.07	0.63
0.027	220	53.68	1.14	0.224	115.93	0.276	128.69	0.82
0.027	240	55.69	1.02	0.39	152.97	0.461	166.31	1.02
0.027	260	60.02	0.81	0.862	227.42	1.016	246.90	1.23
0.027	280†	–	–	–	–	–	–	–
0.035	200	51.42	1.29	0.099	77.07	0.11	81.24	0.71
0.035	220	52.5	1.22	0.181	104.21	0.167	100.10	0.9
0.035	240	53.86	1.13	0.344	143.67	0.299	133.94	1.12
0.035	260	55.84	1.01	0.629	194.27	0.523	177.14	1.26
0.042	200	50.98	1.33	0.088	72.66	0.076	67.53	0.73
0.042	220	51.97	1.25	0.162	98.59	0.142	92.30	0.91
0.042	240	53.07	1.18	0.281	129.85	0.234	118.49	1.16
0.042	260	54.5	1.09	0.419	158.56	0.357	146.36	1.26
0.058	200	50.38	1.38	0.064	61.97	0.042	50.20	0.77
0.058	220	51.17	1.31	0.123	85.91	0.082	70.14	0.94
0.058	240	52.02	1.25	0.197	108.72	0.139	91.32	1.11
0.058	260	52.93	1.19	0.278	129.15	0.213	113.05	1.24
0.064	200	50.21	1.39	0.06	60.00	0.035	45.83	0.74
0.064	220	50.9	1.33	0.104	78.99	0.069	64.43	0.91
0.064	240	51.75	1.27	0.168	100.40	0.122	85.56	1.09
0.064	260	52.57	1.21	0.255	123.69	0.185	105.36	1.25
0.07	200	50.04	1.4	0.049	54.22	0.027	40.25	0.77
0.07	220	50.69	1.35	0.085	71.41	0.052	55.86	0.93
0.07	240	51.47	1.29	0.139	91.32	0.097	76.29	1.08
0.07	260	52.32	1.23	0.205	110.91	0.142	92.30	1.24
0.075	200	49.97	1.41	0.048	53.67	0.019	33.76	0.65
0.075	220	50.6	1.36	0.081	69.71	0.046	52.54	0.85
0.075	240	51.23	1.31	0.117	83.79	0.082	70.14	1.04
0.075	260	52.04	1.25	0.213	113.05	0.127	87.29	1.18
0.081	220	50.41	1.37	0.058	58.99	0.038	47.75	0.77
0.081	240	51.12	1.32	0.106	79.75	0.065	62.45	0.98

continued on next page

†transition to gas phase

Table S3: List of molecular dynamics simulations with varied $\varepsilon(F)$ - Extension.

$\varepsilon(F)$ in kcal/mol	T in K	edge length in Å	ρ in g/cm^3	$D^{C_6H_{14}}$ in Å^2/ps	$\sqrt{6D^{C_6H_{14}}10\,\text{ns}}$ in Å	$D^{C_6F_{14}}$ in Å^2/ps	$\sqrt{6D^{C_6F_{14}}10\,\text{ns}}$ in Å	max $\left(g^{C_6H_{14}-C_6F_{14}}(r)\right)$
0.081	260	51.78	1.27	0.178	103.34	0.104	78.99	1.13
0.081	280	52.62	1.21	0.284	130.54	0.155	96.44	1.25
0.088	220	50.27	1.39	0.062	60.99	0.025	38.73	0.69
0.088	240	50.9	1.33	0.105	79.37	0.051	55.32	0.93
0.088	260	51.6	1.28	0.168	100.40	0.087	72.25	1.1
0.088	280	52.31	1.23	0.253	123.21	0.147	93.91	1.2
0.1	240	50.59	1.36	0.087	72.25	0.032	43.82	0.74
0.1	260	51.2	1.31	0.126	86.95	0.064	61.97	0.97
0.1	280	51.87	1.26	0.188	106.21	0.097	76.29	1.12
0.1	300	52.63	1.21	0.263	125.62	0.15	94.87	1.23
0.1	200*	49.53	1.45	–	–	–	–	–
0.1	220*	50.05	1.4	–	–	–	–	–
0.1	270*	51.53	1.29	–	–	–	–	–
0.1	290*	52.22	1.24	–	–	–	–	–
0.1	310*	53.04	1.18	–	–	–	–	–
0.113	260	50.91	1.33	0.098	76.68	0.04	48.99	0.8
0.113	280	51.48	1.29	0.152	95.50	0.071	65.27	0.96
0.113	300	52.19	1.24	0.233	118.24	0.114	82.70	1.1
0.125	260	50.65	1.35	0.098	76.68	0.021	35.50	0.62
0.125	280	51.22	1.31	0.135	90.00	0.042	50.20	0.77
0.125	300	51.84	1.26	0.195	108.17	0.071	65.27	0.92

*Additional simulation for configurational entropy of mixing calculation

Variation of $\varepsilon(C_H)$

Table S4: List of molecular dynamics simulations performed on a C_6H_{14}-C_6F_{14} mixture (molar fraction $x = 0.5$) with varied energy parameter $\varepsilon(C_H)$. The obtained self-diffusion coefficients, the characteristic diffusive length scales and the height of the first peak in the pair correlation function between the centers of mass of the hexane and perfluorohexane molecules are also provided.

$\varepsilon(C_H)$ in kcal/mol	T in K	edge length in Å	ρ in g/cm^3	$D^{C_6H_{14}}$ in Å^2/ps	$\sqrt{6D^{C_6H_{14}}10\,\text{ns}}$ in Å	$D^{C_6F_{14}}$ in Å^2/ps	$\sqrt{6D^{C_6F_{14}}10\,\text{ns}}$ in Å	max $\left(g^{C_6H_{14}-C_6F_{14}}(r)\right)$
0.012	200	58.68	0.87	1.167	264.61	0.138	90.99	0.63
0.012	220†	–	–	–	–	–	–	–
0.026	200	52.84	1.19	0.294	132.82	0.135	90.00	0.93
0.026	220	54.51	1.09	0.46	166.13	0.228	116.96	1.06
0.026	240	56.99	0.95	0.774	215.50	0.41	156.84	1.18
0.039	200	51.78	1.27	0.171	101.29	0.102	78.23	0.97
0.039	220	52.91	1.19	0.276	128.69	0.169	100.70	1.11
0.039	240	54.32	1.1	0.446	163.58	0.286	131.00	1.21
0.053	200	51.03	1.32	0.113	82.34	0.077	67.97	0.93
0.053	220	51.95	1.26	0.205	110.91	0.124	86.26	1.08
0.053	240	53.03	1.18	0.289	131.68	0.218	114.37	1.22
0.07	200	50.43	1.37	0.061	60.50	0.047	53.10	0.71
0.07	220	51.2	1.31	0.121	85.21	0.089	73.08	0.94
0.07	240	52.06	1.25	0.186	105.64	0.148	94.23	1.14
0.07	260	53.1	1.18	0.297	133.49	0.229	117.22	1.25
0.073	200	50.34	1.38	0.063	61.48	0.045	51.96	0.69
0.073	220	51.12	1.32	0.104	78.99	0.075	67.08	0.85
0.073	240	51.97	1.25	0.177	103.05	0.143	92.63	1.09
0.073	260	52.92	1.19	0.293	132.59	0.203	110.36	1.23
0.08	200	50.18	1.39	0.047	53.10	0.039	48.37	0.61
0.08	220	50.89	1.34	0.075	67.08	0.07	64.81	0.78
0.08	240	51.67	1.28	0.149	94.55	0.117	83.79	1.04
0.08	260	52.55	1.21	0.226	116.45	0.192	107.33	1.15
0.08	280	53.69	1.14	0.361	147.17	0.277	128.92	1.29
0.093	200	49.88	1.42	0.028	40.99	0.031	43.13	0.5
0.093	220	50.5	1.37	0.053	56.39	0.063	61.48	0.6
0.093	240	51.26	1.31	0.096	75.89	0.098	76.68	0.76
0.093	260	52.07	1.25	0.174	102.18	0.151	95.18	1.04
0.093	280	53.02	1.18	0.289	131.68	0.237	119.25	1.16
0.093	300	54.18	1.11	0.408	156.46	0.349	144.71	1.26
0.093	230*	50.87	1.34	–	–	–	–	–
0.093	250*	51.64	1.28	–	–	–	–	–

continued on next page

† transition to gas phase

* Additional simulation for configurational entropy of mixing calculation

Table S4: List of molecular dynamics simulations with varied $\varepsilon(C_H)$ - Extension.

$\varepsilon(C_H)$ in kcal/mol	T in K	edge length in Å	ρ in g/cm^3	$D^{C_6H_{14}}$ in Å^2/ps	$\sqrt{6D^{C_6H_{14}}10\,\text{ns}}$ in Å	$D^{C_6F_{14}}$ in Å^2/ps	$\sqrt{6D^{C_6F_{14}}10\,\text{ns}}$ in Å	max $\left(g^{C_6H_{14}-C_6F_{14}}(r)\right)$
0.093	270*	52.54	1.21	–	–	–	–	–
0.1	220	50.39	1.38	0.041	49.60	0.057	58.48	0.57
0.1	240	51.1	1.32	0.075	67.08	0.086	71.83	0.68
0.1	260	51.85	1.26	0.124	86.26	0.132	88.99	0.84
0.1	280	52.75	1.2	0.212	112.78	0.219	114.63	0.98
0.1	300	53.77	1.13	0.325	139.64	0.292	132.36	1.17
0.11	220	50.2	1.39	0.032	43.82	0.044	51.38	0.5
0.11	240	50.88	1.34	0.053	56.39	0.086	71.83	0.59
0.11	260	51.59	1.28	0.089	73.08	0.109	80.87	0.73
0.11	280	52.4	1.22	0.158	97.37	0.176	102.76	0.86
0.11	300	53.41	1.16	0.265	126.10	0.269	127.04	0.97
0.12	200	49.5	1.45	0.012	26.83	0.024	37.95	0.41
0.12	220	50.07	1.4	0.026	39.50	0.042	50.20	0.45
0.12	240	50.7	1.35	0.045	51.96	0.073	66.18	0.56
0.12	260	51.39	1.3	0.069	64.34	0.109	80.87	0.65
0.12	280	52.17	1.24	0.102	78.23	0.158	97.37	0.74
0.12	300	53.11	1.17	0.199	109.27	0.243	120.75	0.83
0.147	220	49.79	1.43	0.012	26.83	0.037	41.12	0.41
0.147	240	50.37	1.38	0.021	35.50	0.057	58.48	0.46
0.147	260	50.92	1.33	0.032	43.82	0.084	70.99	0.51
0.147	280	51.67	1.28	0.058	58.99	0.132	88.99	0.58
0.147	300	52.53	1.21	0.069	64.34	0.203	110.36	0.61

*Additional simulation for configurational entropy of mixing calculation

Variation of $\varepsilon(C_F)$

Table S5: List of molecular dynamics simulations performed on a C_6H_{14}-C_6F_{14} mixture (molar fraction $x = 0.5$) with varied energy parameter $\varepsilon(C_F)$. The obtained self-diffusion coefficients, the characteristic diffusive length scales and the height of the first peak in the pair correlation function between the centers of mass of the hexane and perfluorohexane molecules are also provided.

$\varepsilon(C_F)$ in kcal/mol	T in K	edge length in Å	ρ in g/cm^3	$D^{C_6H_{14}}$ in Å^2/ps	$\sqrt{6D^{C_6H_{14}}10\,\mathrm{ns}}$ in Å	$D^{C_6F_{14}}$ in Å^2/ps	$\sqrt{6D^{C_6F_{14}}10\,\mathrm{ns}}$ in Å	max $\left(g^{C_6H_{14}-C_6F_{14}}(r)\right)$
0.012	200	52.21	1.06	0.116	83.43	0.325	139.64	0.54
0.012	220	58.15	0.9	0.26	124.90	0.586	187.51	0.66
0.012	240†	–	–	–	–	–	–	–
0.026	200	52.51	1.22	0.113	82.34	0.148	94.23	0.64
0.026	220	53.88	1.13	0.211	112.52	0.26	124.90	0.79
0.026	240	55.75	1.02	0.376	150.20	0.388	152.58	1.03
0.026	260	58.9	0.86	0.679	201.84	0.735	210.00	1.18
0.039	200	51.54	1.29	0.101	77.85	0.094	75.10	0.69
0.039	220	52.64	1.21	0.186	105.64	0.166	99.80	0.88
0.039	240	53.91	1.12	0.316	137.70	0.27	127.28	1.11
0.039	260	55.78	1.01	0.532	178.66	0.442	162.85	1.24
0.053	200	50.96	1.33	0.089	73.08	0.069	64.34	0.73
0.053	220	51.87	1.26	0.155	96.44	0.131	88.66	0.97
0.053	240	52.92	1.19	0.268	126.81	0.212	112.78	1.15
0.053	260	54.22	1.1	0.414	157.61	0.298	133.72	1.28
0.08	200	50.17	1.39	0.058	58.99	0.038	47.75	0.73
0.08	220	50.92	1.33	0.116	83.43	0.077	67.97	0.96
0.08	240	51.72	1.27	0.177	103.05	0.121	85.21	1.16
0.08	260	52.67	1.21	0.28	129.61	0.21	112.25	1.26
0.093	200	49.9	1.42	0.049	54.22	0.026	39.50	0.71
0.093	220	50.56	1.36	0.094	75.10	0.057	58.48	0.9
0.093	240	51.33	1.3	0.155	96.44	0.105	79.37	1.1
0.093	260	52.13	1.24	0.237	119.25	0.156	96.75	1.24
0.093	280	53.12	1.17	0.342	143.25	0.229	117.22	1.31
0.107	200	49.63	1.44	0.045	51.96	0.021	35.50	0.66
0.107	220	50.24	1.39	0.082	70.14	0.05	54.77	0.95
0.107	240	50.94	1.33	0.117	83.79	0.077	67.97	1.08
0.107	260	51.71	1.27	0.203	110.36	0.134	89.67	1.22
0.107	280	52.58	1.21	0.282	130.08	0.188	106.21	1.31
0.12	200	49.42	1.46	0.039	48.37	0.016	30.98	0.6
0.12	220	49.98	1.41	0.062	60.99	0.034	45.17	0.83
0.12	240	50.64	1.36	0.116	84.43	0.067	63.40	1.03
0.12	260	51.35	1.3	0.175	102.47	0.104	78.99	1.2

continued on next page

† transition to gas phase

Table S5: List of molecular dynamics simulations with varied $\varepsilon(C_F)$ - Extension.

$\varepsilon(C_F)$ in kcal/mol	T in K	edge length in Å	ρ in g/cm^3	$D^{C_6H_{14}}$ in Å^2/ps	$\sqrt{6D^{C_6H_{14}}10\,\text{ns}}$ in Å	$D^{C_6F_{14}}$ in Å^2/ps	$\sqrt{6D^{C_6F_{14}}10\,\text{ns}}$ in Å	max $\left(g^{C_6H_{14}-C_6F_{14}}(r)\right)$
0.12	280	52.15	1.24	0.256	123.94	0.161	98.29	1.29
0.134	220	49.74	1.43	0.061	60.50	0.024	37.95	0.69
0.134	240	50.4	1.38	0.092	74.30	0.052	55.86	0.96
0.134	260	51.03	1.32	0.141	91.98	0.09	73.48	1.13
0.134	280	51.75	1.27	0.23	117.47	0.134	89.67	1.27
0.147	220	49.59	1.44	0.054	56.92	0.018	32.86	0.64
0.147	240	50.15	1.4	0.083	70.57	0.044	51.38	0.92
0.147	260	50.75	1.35	0.133	89.33	0.071	65.27	1.06
0.147	280	51.43	1.29	0.196	108.44	0.121	85.21	1.23
0.147	300	52.23	1.24	0.292	132.36	0.178	103.34	1.31
0.161	220	49.36	1.46	0.052	55.86	0.013	27.93	0.6
0.161	240	49.9	1.42	0.07	64.81	0.026	39.50	0.74
0.161	260	50.53	1.37	0.122	85.56	0.059	59.50	1.03
0.161	280	51.17	1.31	0.181	104.21	0.092	74.30	1.14
0.161	300	51.84	1.26	0.268	126.81	0.141	91.98	1.26
0.174	240	49.72	1.43	0.07	64.81	0.02	34.64	0.64
0.174	260	50.28	1.38	0.107	80.12	0.042	50.20	0.87
0.174	280	50.92	1.33	0.16	97.98	0.076	67.53	1.06
0.174	300	51.57	1.28	0.222	115.41	0.106	79.75	1.17
0.201	260	49.95	1.41	0.101	77.85	0.025	38.73	0.63
0.201	280	50.49	1.37	0.126	86.95	0.045	51.96	0.84
0.201	300	51.07	1.32	0.191	107.05	0.078	68.41	1.03

1.3 Simulations with modified Lennard-Jones size parameters

Variation of $\sigma(H)$

Table S6: List of molecular dynamics simulations performed on a C_6H_{14}-C_6F_{14} mixture (molar fraction $x = 0.5$) with varied size parameter $\sigma(H)$. The obtained self-diffusion coefficients, the characteristic diffusive length scales and the height of the first peak in the pair correlation function between the centers of mass of the hexane and perfluorohexane molecules are also provided.

$\sigma(H)$ in Å	T in K	edge length in Å	ρ in g/cm^3	$D^{C_6H_{14}}$ in Å^2/ps	$\sqrt{6D^{C_6H_{14}}10\,\text{ns}}$ in Å	$D^{C_6F_{14}}$ in Å^2/ps	$\sqrt{6D^{C_6F_{14}}10\,\text{ns}}$ in Å	max $\left(g^{C_6H_{14}-C_6F_{14}}(r)\right)$
1.87	200	49.08	1.489	0.0977	76.56	0.0605	60.25	1.09
1.87	220	49.8	1.426	0.1655	99.65	0.0977	76.56	1.21
2	200	49.23	1.476	0.0919	74.26	0.0538	56.82	0.94
2	220	49.94	1.414	0.1424	92.43	0.0861	71.87	1.16
2	240	50.76	1.347	0.2486	122.13	0.1462	93.66	1.27
2	260	51.67	1.276	0.3233	139.28	0.2081	111.74	1.35
2.12	200	49.44	1.46	0.0768	67.88	0.0555	57.71	0.88
2.12	220	50.16	1.395	0.1279	87.60	0.0935	74.90	1.08
2.12	240	50.98	1.329	0.2072	111.50	0.139	91.32	1.24
2.25	200	49.75	1.43	0.0747	66.95	0.0557	57.81	0.87
2.25	220	50.52	1.366	0.1367	90.56	0.1006	77.69	1.06
2.25	240	51.35	1.301	0.2041	110.66	0.1458	93.53	1.2
2.37	200	50.11	1.4	0.0699	64.76	0.0479	53.61	0.82
2.37	220	50.87	1.338	0.1282	87.70	0.0972	76.37	1.03
2.37	240	51.77	1.269	0.2178	114.32	0.1505	95.03	1.17
2.62	200	51.01	1.327	0.0747	66.95	0.0543	57.08	0.75
2.62	220	51.84	1.264	0.1345	89.83	0.104	78.99	0.96
2.62	240	52.807	1.196	0.2248	116.14	0.1728	101.82	1.13
2.62	260	53.93	1.123	0.3583	146.62	0.2725	127.878	1.26
2.75	200	51.59	1.282	0.0859	71.79	0.062	60.99	0.74
2.75	220	52.46	1.22	0.1498	94.81	0.1131	82.38	0.95
2.75	240	53.49	1.15	0.233	118.24	0.1782	103.40	1.11
2.75	260	54.7	1.076	0.3996	154.84	0.2852	130.81	1.25
2.87	200	52.13	1.243	0.0843	71.12	0.0657	62.79	0.72
2.87	220	53.1	1.176	0.1601	98.01	0.1212	85.28	0.91
2.87	240	54.15	1.109	0.2846	130.68	0.205	110.91	1.07
2.87	260	55.43	1.031	0.4506	164.43	0.3083	136.01	1.21
3	200	52.78	1.198	0.1082	80.57	0.0618	60.89	0.67
3	220	53.79	1.132	0.1917	107.25	0.1241	86.29	0.89
3	240	54.98	1.06	0.3109	136.58	0.2072	111.50	1.04
3	260	56.48	0.978	0.4889	171.27	0.3568	146.31	1.17
3.12	200	53.49	1.151	0.1194	84.64	0.0911	73.93	0.77
3.12	220	54.5	1.088	0.1961	108.47	0.1399	91.62	0.9
3.12	240	55.77	1.015	0.3428	143.42	0.2224	115.52	1.01

continued on next page

Table S6: List of molecular dynamics simulations with varied $\sigma(H)$ - Extension.

$\sigma(H)$ in Å	T in K	edge length in Å	ρ in g/cm^3	$D^{C_6H_{14}}$ in Å^2/ps	$\sqrt{6D^{C_6H_{14}}10\,\text{ns}}$ in Å	$D^{C_6F_{14}}$ in Å^2/ps	$\sqrt{6D^{C_6F_{14}}10\,\text{ns}}$ in Å	max $\left(g^{C_6H_{14}-C_6F_{14}}(r)\right)$
3.12	260	57.39	0.93	0.5545	182.40	0.3991	154.74	1.15
3.25	220	55.38	1.04	0.2405	120.12	0.1495	94.71	0.89
3.25	240	56.73	0.94	0.3936	153.67	0.262	125.38	1.01
3.25	260	58.61	0.87	0.6374	195.56	0.4305	160.72	1.11
3.25	280	61.58	0.75	1.0345	249.14	0.73	209.28	1.21
3.37	220	56.21	0.991	0.2651	126.12	0.1646	99.38	0.88
3.37	240	57.64	0.92	0.4266	159.99	0.2891	131.70	1.03
3.37	260	59.84	0.82	0.716	207.27	0.52	176.64	1.11
3.37	280	63.94	0.674	1.3543	285.06	0.8344	223.75	1.19
3.5	200	55.98	1.004	0.1766	102.94	0.0792	68.93	0.7
3.5	220	57.21	0.94	0.288	131.45	0.171	101.29	0.84
3.5	240	58.84	0.864	0.5022	173.59	0.3321	141.16	1.02
3.5	260	61.5	0.757	0.8934	231.53	0.6019	190.04	1.1
3.5	280†	–	–	–	–	–	–	–
3.62	200	56.82	0.96	0.1888	106.43	0.0982	76.76	0.68
3.62	220	58.19	0.893	0.3621	147.40	0.1886	106.38	0.85
3.62	240	60.22	0.806	0.5872	187.70	0.3704	149.08	1.01
3.62	260	63.37	0.69	1.009	246.05	0.7224	208.19	1.13
3.62	280†	–	–	–	–	–	–	–
3.75	200	57.91	0.907	0.2452	121.29	0.1104	81.39	0.65
3.75	220	59.6	0.832	0.4261	159.89	0.2391	119.77	0.87
3.75	240	61.75	0.748	0.7328	209.69	0.4057	156.02	1.03
3.75	260	66.34	0.603	1.3048	279.80	0.9271	235.85	1.14
3.75	280†	–	–	–	–	–	–	–
3.75	300†	–	–	–	–	–	–	–

† transition to gas phase

Variation of $\sigma(F)$

Table S7: List of molecular dynamics simulations performed on a C_6H_{14}-C_6F_{14} mixture (molar fraction $x = 0.5$) with varied size parameter $\sigma(F)$. The obtained self-diffusion coefficients, the characteristic diffusive length scales and the height of the first peak in the pair correlation function between the centers of mass of the hexane and perfluorohexane molecules are also provided.

$\sigma(F)$ in Å	T in K	edge length in Å	ρ in g/cm^3	$D^{C_6H_{14}}$ in Å^2/ps	$\sqrt{6D^{C_6H_{14}}10\,\text{ns}}$ in Å	$D^{C_6F_{14}}$ in Å^2/ps	$\sqrt{6D^{C_6F_{14}}10\,\text{ns}}$ in Å	max $\left(g^{C_6H_{14}-C_6F_{14}}(r)\right)$
2.45	200	47.97	1.61	0.054	56.92	0.034	45.17	0.81
2.45	220	48.59	1.54	0.095	75.50	0.067	63.40	1.14
2.45	240	49.31	1.47	0.158	97.37	0.121	85.21	1.31
2.45	260	50.13	1.4	0.243	120.75	0.175	102.47	1.39
2.7	200	49.16	1.48	0.063	61.48	0.0435	51.09	0.79
2.7	220	49.91	1.416	0.109	80.87	0.0825	70.36	1.04
2.7	240	50.75	1.35	0.189	106.49	0.129	87.98	1.25
3.08	220	52.21	1.23	0.1519	220.45	0.107	184.61	0.91
3.08	240	53.16	1.17	0.2377	119.42	0.1836	104.96	1.11
3.2	200	52.13	1.243	0.0845	71.20	0.0646	62.26	0.77
3.2	220	52.99	1.183	0.1617	98.50	0.1173	83.89	0.96
3.2	240	53.99	1.119	0.2679	126.78	0.1797	103.84	1.11
3.2	260	55.26	1.044	0.3872	152.42	0.3124	136.91	1.18
3.33	220	53.93	1.12	0.162	98.59	0.1323	89.10	0.93
3.33	240	55.03	1.06	0.2836	130.45	0.2116	112.68	1.09
3.45	200	53.93	1.12	0.099	77.07	0.084	70.99	0.78
3.45	220	54.8	1.07	0.191	107.05	0.1497	94.77	0.97
3.45	240	56.01	1	0.323	139.21	0.236	119.00	1.08
3.45	260	57.44	0.93	0.469	167.75	0.363	147.58	1.18
3.58	220	55.93	1.01	0.2147	113.50	0.1662	99.86	0.94
3.58	240	57.18	0.94	0.3587	146.70	0.2449	121.22	1.05
3.7	200	55.91	1.01	0.0999	77.42	0.104	78.99	0.68
3.7	220	57.02	0.95	0.225	116.19	0.183	104.79	0.92
3.7	240	58.3	0.89	0.354	145.74	0.287	131.22	1.09
3.7	260	60.22	0.81	0.65	197.48	0.4575	165.68	1.2
3.95	200	58.25	0.89	0.121	85.21	0.141	91.98	0.7
3.95	220	59.55	0.83	0.268	126.81	0.255	123.69	0.91
3.95	240	61.37	0.76	0.4656	167.14	0.4134	157.49	1.12
3.95	260	64.06	0.67	0.8432	224.93	0.647	197.03	1.24
4.2	220	63.31	0.694	0.353	145.53	0.38	151.00	0.87
4.2	240	66.23	0.606	0.606	190.68	0.642	196.27	1.05
4.2	260†	–	–	–	–	–	–	–
4.2	280†	–	–	–	–	–	–	–
4.45	200	66.39	0.602	0.166	99.80	0.4763	169.05	0.69

continued on next page

†transition to gas phase

Table S7: List of molecular dynamics simulations with varied $\sigma(F)$ - Extension.

$\sigma(F)$ in Å	T in K	edge length in Å	ρ in g/cm^3	$D^{C_6H_{14}}$ in Å^2/ps	$\sqrt{6D^{C_6H_{14}} 10\,\text{ns}}$ in Å	$D^{C_6F_{14}}$ in Å^2/ps	$\sqrt{6D^{C_6H_{14}} 10\,\text{ns}}$ in Å	max $\left(g^{C_6H_{14}-C_6F_{14}}(r)\right)$
4.45	220†	–	–	–	–	–	–	–
4.45	240†	–	–	–	–	–	–	–

†transition to gas phase

Variation of $\sigma(C_H)$

Table S8: List of molecular dynamics simulations performed on a C_6H_{14}-C_6F_{14} mixture (molar fraction $x = 0.5$) with varied size parameter $\sigma(C_H)$. The obtained self-diffusion coefficients, the characteristic diffusive length scales and the height of the first peak in the pair correlation function between the centers of mass of the hexane and perfluorohexane molecules are also provided.

$\sigma(C_H)$ in Å	T in K	edge length in Å	ρ in g/cm^3	$D^{C_6H_{14}}$ in Å^2/ps	$\sqrt{6D^{C_6H_{14}}10\,\mathrm{ns}}$ in Å	$D^{C_6F_{14}}$ in Å^2/ps	$\sqrt{6D^{C_6F_{14}}10\,\mathrm{ns}}$ in Å	max $\left(g^{C_6H_{14}-C_6F_{14}}(r)\right)$
2.50	200	57.58	0.92	0.98	242.49	0.132	88.99	0.65
2.50	220†	–	–	–	–	–	–	–
2.63	200	54.29	1.1	0.508	174.59	0.148	94.23	0.75
2.63	220	57.54	0.92	0.9	232.38	0.289	131.68	0.91
2.63	240†	–	–	–	–	–	–	–
2.75	200	53.16	1.17	0.318	138.13	0.125	86.60	0.88
2.75	220	54.99	1.06	0.559	183.14	0.266	126.33	1.03
2.75	240	58.22	0.89	1.016	246.90	0.453	164.86	1.15
2.88	200	52.32	1.23	0.23	117.47	0.126	86.95	0.97
2.88	220	53.8	1.13	0.393	153.56	0.207	111.45	1.1
2.88	240	55.89	1.09	0.676	201.40	0.368	148.59	1.22
3.00	200	51.75	1.27	0.186	105.64	0.102	78.23	0.98
3.00	220	53.03	1.18	0.304	135.06	0.182	104.50	1.12
3.00	240	54.58	1.08	0.529	178.16	0.284	130.54	1.22
3.00	260	57.13	0.94	0.842	224.77	0.517	176.12	1.3
3.13	200	51.31	1.3	0.126	86.95	0.089	73.08	0.99
3.13	220	52.39	1.22	0.233	118.24	0.162	98.59	1.12
3.13	240	53.66	1.14	0.381	151.20	0.245	121.24	1.25
3.25	200	50.99	1.33	0.115	83.07	0.068	63.87	0.89
3.25	220	51.94	1.26	0.204	110.63	0.142	92.30	1.09
3.25	240	53.07	1.18	0.309	136.16	0.212	112.78	1.22
3.38	200	50.69	1.35	0.09	73.48	0.064	61.97	0.83
3.38	220	51.62	1.28	0.15	94.87	0.113	82.34	1.08
3.38	240	52.59	1.21	0.265	126.10	0.177	103.05	1.19
3.63	200	50.44	1.37	0.054	56.92	0.044	51.38	0.71
3.63	220	51.18	1.31	0.104	78.99	0.082	70.14	0.94
3.63	240	52.04	1.25	0.181	104.21	0.142	92.30	1.13
3.63	260	52.99	1.18	0.277	128.92	0.213	113.05	1.25
3.75	200	50.47	1.37	0.056	57.97	0.043	50.79	0.7
3.75	220	51.12	1.32	0.091	73.89	0.076	67.53	0.85
3.75	240	51.91	1.26	0.169	100.70	0.127	87.29	1.04
3.75	260	52.84	1.19	0.243	120.75	0.184	105.07	1.22
3.88	200	50.52	1.36	0.048	53.67	0.039	48.37	0.71

continued on next page

† transition to gas phase

Table S8: List of molecular dynamics simulations with varied $\sigma(C_H)$ - Extension.

$\sigma(C_H)$ in Å	T in K	edge length in Å	ρ in g/cm^3	$D^{C_6H_{14}}$ in Å^2/ps	$\sqrt{6D^{C_6H_{14}}10\,\mathrm{ns}}$ in Å	$D^{C_6F_{14}}$ in Å^2/ps	$\sqrt{6D^{C_6F_{14}}10\,\mathrm{ns}}$ in Å	max $\left(g^{C_6H_{14}-C_6F_{14}}(r)\right)$
3.88	220	51.18	1.31	0.089	73.08	0.071	65.27	0.88
3.88	240	51.89	1.26	0.15	94.87	0.119	84.50	1.08
3.88	260	52.74	1.2	0.234	118.49	0.19	106.77	1.2
4.00	200	50.66	1.35	0.051	55.32	0.041	49.60	0.81
4.00	220	51.229	1.3	0.091	73.89	0.073	66.18	0.96
4.00	240	51.96	1.25	0.134	89.67	0.115	83.07	1.06
4.00	260	52.75	1.2	0.21	112.25	0.178	103.34	1.17
4.00	280	53.66	1.14	0.34	142.83	0.263	125.62	1.24
4.13	200	50.85	1.34	0.054	56.92	0.042	50.20	0.9
4.13	220	51.47	1.29	0.095	75.50	0.075	67.08	1.01
4.13	240	52.12	1.24	0.152	95.50	0.118	84.14	1.12
4.13	260	52.83	1.19	0.218	114.37	0.176	102.76	1.18
4.13	280	53.7	1.14	0.317	137.91	0.244	121.00	1.22
4.25	200	51.08	1.32	0.055	57.45	0.046	52.54	1.12
4.25	220	51.67	1.28	0.106	79.75	0.078	68.41	1.09
4.25	240	52.31	1.23	0.167	100.10	0.11	81.24	1.17
4.25	260	53	1.18	0.23	117.47	0.177	103.05	1.19
4.25	280	53.82	1.13	0.321	138.78	0.241	120.25	1.23
4.38	200	51.35	1.3	0.059	59.50	0.045	51.96	1.3
4.38	220	51.93	1.26	0.106	79.75	0.078	68.41	1.23
4.38	240	52.57	1.21	0.148	94.23	0.132	88.99	1.17
4.38	260	53.24	1.17	0.229	117.22	0.181	104.21	1.21
4.5	200	51.66	1.28	0.054	56.92	0.047	53.10	1.6
4.5	220	52.21	1.24	0.101	77.85	0.077	67.97	1.33
4.5	240	52.82	1.19	0.163	98.89	0.118	84.14	1.26
4.5	260	53.53	1.15	0.244	121.00	0.171	101.29	1.24

Variation of $\sigma(C_F)$

Table S9: List of molecular dynamics simulations performed on a C_6H_{14}-C_6F_{14} mixture (molar fraction $x = 0.5$) with varied size parameter $\sigma(C_F)$. The obtained self-diffusion coefficients, the characteristic diffusive length scales and the height of the first peak in the pair correlation function between the centers of mass of the hexane and perfluorohexane molecules are also provided.

$\sigma(C_F)$ in Å	T in K	edge length in Å	ρ in g/cm^3	$D^{C_6H_{14}}$ in Å^2/ps	$\sqrt{6D^{C_6H_{14}}10\,\text{ns}}$ in Å	$D^{C_6F_{14}}$ in Å^2/ps	$\sqrt{6D^{C_6F_{14}}10\,\text{ns}}$ in Å	max $\left(g^{C_6H_{14}-C_6F_{14}}(r)\right)$
2.25	200	57.94	0.9	0.104	78.99	0.573	185.42	0.52
2.25	220†	–	–	–	–	–	–	–
2.37	200	56.54	0.97	0.107	80.12	0.425	159.69	0.52
2.37	220†	–	–	–	–	–	–	–
2.5	200	55.17	1.05	0.098	76.68	0.344	143.67	0.54
2.5	220	59.21	0.85	0.23	117.47	0.71	206.40	0.67
2.5	240†	–	–	–	–	–	–	–
2.5	260†	–	–	–	–	–	–	–
2.62	200	54.07	1.11	0.111	81.61	0.23	117.47	0.58
2.62	220	56.71	0.96	0.234	118.49	0.453	164.86	0.69
2.62	240†	–	–	–	–	–	–	–
2.75	200	53.25	1.17	0.106	79.75	0.186	105.64	0.58
2.75	220	55	1.06	0.216	113.84	0.321	138.78	0.73
2.75	240	58.36	0.88	0.424	159.50	0.699	204.79	0.94
2.75	260†	–	–	–	–	–	–	–
2.87	200	52.58	1.21	0.1	77.46	0.155	96.44	0.61
2.87	220	53.99	1.12	0.188	106.21	0.263	125.62	0.77
2.87	240	56.2	0.99	0.373	149.60	0.436	161.74	0.97
2.87	260	60.6	0.79	0.834	223.70	0.909	233.54	1.18
3.00	200	52.03	1.25	0.095	75.50	0.13	88.32	0.65
3.00	220	53.25	1.17	0.179	103.63	0.208	111.71	0.83
3.00	240	54.82	1.07	0.373	149.60	0.34	142.83	1.1
3.00	260	57.44	0.93	0.699	204.79	0.619	192.72	1.24
3.13	200	51.54	1.29	0.092	74.30	0.094	75.10	0.67
3.13	220	52.63	1.21	0.179	103.63	0.169	100.70	0.92
3.13	240	53.92	1.12	0.305	135.28	0.268	126.81	1.11
3.13	260	55.77	1.01	0.517	176.12	0.438	162.11	1.27
3.25	200	51.17	1.31	0.09	73.48	0.082	70.14	0.72
3.25	220	52.11	1.24	0.167	100.10	0.139	91.32	0.95
3.25	240	53.29	1.16	0.298	133.72	0.233	118.24	1.14
3.25	260	54.81	1.07	0.432	161.00	0.369	148.80	1.29
3.37	200	50.87	1.34	0.084	70.99	0.067	63.40	0.77
3.37	220	51.74	1.27	0.141	91.98	0.119	84.50	0.96

continued on next page

†transition to gas phase

Table S9: List of molecular dynamics simulations with varied $\sigma(C_F)$ - Extension.

$\sigma(C_F)$ in Å	T in K	edge length in Å	ρ in g/cm^3	$D^{C_6H_{14}}$ in Å^2/ps	$\sqrt{6D^{C_6H_{14}}10\,\text{ns}}$ in Å	$D^{C_6F_{14}}$ in Å^2/ps	$\sqrt{6D^{C_6F_{14}}10\,\text{ns}}$ in Å	max $\left(g^{C_6H_{14}-C_6F_{14}}(r)\right)$
3.37	240	52.72	1.2	0.211	112.52	0.199	109.27	1.15
3.37	260	54.01	1.11	0.386	152.18	0.306	135.50	1.3
3.62	200	50.33	1.38	0.06	60.00	0.044	51.38	0.71
3.62	220	51.07	1.32	0.118	84.14	0.078	68.41	0.98
3.62	240	51.87	1.26	0.184	105.07	0.131	88.66	1.15
3.62	260	52.84	1.19	0.291	132.14	0.2	109.54	1.27
3.75	200	50.09	1.4	0.058	58.99	0.034	45.17	0.8
3.75	220	50.79	1.34	0.101	77.85	0.063	61.48	0.94
3.75	240	51.56	1.28	0.168	100.40	0.118	84.14	1.09
3.75	260	52.38	1.22	0.246	121.49	0.169	100.70	1.21
3.87	200	49.94	1.41	0.049	54.22	0.027	40.25	0.75
3.87	220	50.59	1.36	0.096	75.89	0.06	60.00	0.95
3.87	240	51.32	1.3	0.141	91.98	0.093	74.70	1.1
3.87	260	52.04	1.25	0.22	114.89	0.139	91.32	1.2
4.00	200	49.84	1.42	0.043	50.79	0.021	35.50	0.74
4.00	220	50.41	1.37	0.069	64.34	0.043	50.79	0.93
4.00	240	51.1	1.32	0.133	89.33	0.091	73.89	1.08
4.00	260	51.78	1.27	0.196	108.44	0.132	88.99	1.17
4.00	280	52.56	1.21	0.29	131.91	0.191	107.05	1.27
4.12	200	49.78	1.43	0.039	48.37	0.018	32.86	0.76
4.12	220	50.37	1.38	0.067	63.40	0.037	47.12	0.89
4.12	240	51	1.33	0.122	85.56	0.074	66.63	1.06
4.12	260	51.61	1.28	0.175	102.47	0.11	81.24	1.15
4.12	280	52.31	1.23	0.247	121.74	0.161	98.29	1.23
4.25	200	49.78	1.43	0.037	47.12	0.015	30.00	0.77
4.25	220	50.31	1.38	0.064	61.97	0.034	45.17	0.93
4.25	240	50.87	1.34	0.104	78.99	0.062	60.99	1.06
4.25	260	51.48	1.29	0.156	96.75	0.101	77.85	1.14
4.25	280	52.11	1.24	0.207	111.45	0.157	97.06	1.21
4.37	200	49.81	1.42	0.035	45.83	0.014	28.98	0.75
4.37	220	50.3	1.38	0.062	60.99	0.032	43.82	0.96
4.37	240	50.87	1.34	0.097	76.29	0.058	58.99	1.06
4.37	260	51.41	1.3	0.149	94.55	0.088	72.66	1.14
4.37	280	52.04	1.25	0.229	117.22	0.134	89.67	1.2
4.5	200	49.91	1.42	0.029	41.71	0.013	27.93	0.9
4.5	220	50.38	1.38	0.054	56.92	0.03	42.43	1.02
4.5	240	50.91	1.33	0.093	74.70	0.055	57.45	1.08
4.5	260	51.42	1.29	0.149	94.55	0.088	72.66	1.15
4.5	280	52	1.25	0.194	107.89	0.127	87.29	1.2

continued on next page

† transition to gas phase

Table S9: List of molecular dynamics simulations with varied $\sigma(C_F)$ - Extension.

$\sigma(C_F)$ in Å	T in K	edge length in Å	ρ in g/cm^3	$D^{C_6H_{14}}$ in Å^2/ps	$\sqrt{6D^{C_6H_{14}}10\,\mathrm{ns}}$ in Å	$D^{C_6F_{14}}$ in Å^2/ps	$\sqrt{6D^{C_6F_{14}}10\,\mathrm{ns}}$ in Å	max $\left(g^{C_6H_{14}-C_6F_{14}}(r)\right)$
4.62	200	50.04	1.4	0.027	40.25	0.011	25.69	0.99
4.62	220	50.53	1.36	0.052	55.86	0.032	43.82	1.09
4.62	240	50.99	1.33	0.088	72.66	0.05	54.77	1.12
4.62	260	51.49	1.29	0.137	90.66	0.084	70.99	1.16

2 Temperature dependence of self-diffusion coefficients

2.1 Arrhenius type analysis

For each set of force field parameters, the temperature dependence of the self-diffusion coefficients (Tables S1-S9) was further analyzed. Data was plotted in Arrhenius plots and fitted with Equation 1 to obtain diffusion activation energies, E_A, for hexane and perfluorohexane molecules as well as the pre-exponential factors, D_0. An example, the corresponding Arrhenius plots for the variation of $\varepsilon(F)$, is shown in Figure S1.

$$\ln D(T) = \ln D_0 - \frac{E_A}{RT} \tag{1}$$

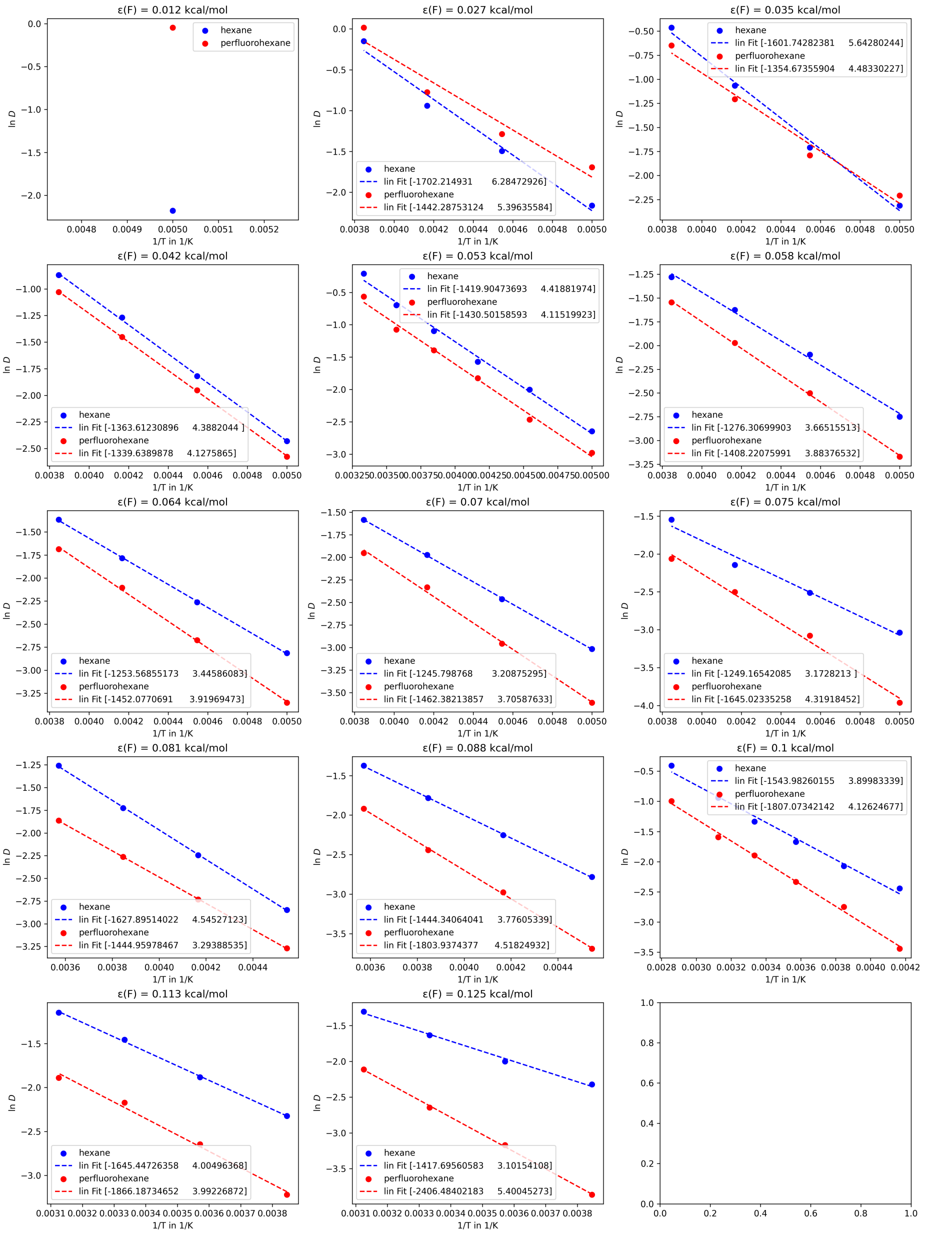

Figure S1: Arrhenius plots of the self-diffusion coefficients of hexane (blue points) and perfluorohexane (red points) in a binary mixture ($x = 0.5$) dependent on the value of the Lennard-Jones energy parameter $\varepsilon(F)$. Dashed lines correspond to linear fits with Equation 1. The fit coefficients in square brackets equal $-\frac{E_A}{k_B N_A}$ and $\ln(D_0)$.

For the variation of all other parameters ($\varepsilon(H)$, $\varepsilon(C_H)$, $\varepsilon(C_F)$, $\sigma(H)$, $\sigma(F)$, $\sigma(C_H)$ and $\sigma(C_F)$), the same procedure was followed. The resulting diffusion activation energies and pre-exponential factors are compiled in Table S10.

Table S10: Activation energies and pre-exponential factors for hexane and perfluorohexane self-diffusion in a binary mixture ($x = 0.5$). Obtained from an Arrhenius type analysis.

parameter set	$E_A^{C_6H_{14}}$ in kJ/mol	$D_0^{C_6H_{14}}$ in Å^2/ps	$E_A^{C_6F_{14}}$ in kJ/mol	$D_0^{C_6F_{14}}$ in Å^2/ps
OPLS-AA	11.8	36.7	11.89	61.26
$\varepsilon(H)$ =0.008 kcal/mol	8.63	45.46	10.38	76.73
$\varepsilon(H)$ =0.015 kcal/mol	9.88	60.09	9.07	24.48
$\varepsilon(H)$ =0.023 kcal/mol	10.4	56.91	10.36	36.7
$\varepsilon(H)$ =0.038 kcal/mol	13.07	103.24	11.71	44.33
$\varepsilon(H)$ =0.045 kcal/mol	14.4	136.41	12.15	46.83
$\varepsilon(H)$ =0.053 kcal/mol	15.83	166.67	12.55	44.94
$\varepsilon(H)$ =0.06 kcal/mol	15.08	80.33	14.01	73.29
$\varepsilon(F)$ =0.027 kcal/mol	14.15	536.46	11.99	220.52
$\varepsilon(F)$ =0.035 kcal/mol	13.31	282.31	11.26	88.5
$\varepsilon(F)$ =0.042 kcal/mol	11.33	80.48	11.13	62.05
$\varepsilon(F)$ =0.058 kcal/mol	10.61	39.06	11.7	48.57
$\varepsilon(F)$ =0.064 kcal/mol	10.42	31.37	12.07	50.4
$\varepsilon(F)$ =0.07 kcal/mol	10.35	24.75	12.15	40.69
$\varepsilon(F)$ =0.075 kcal/mol	10.38	23.88	13.67	75.11
$\varepsilon(F)$ =0.081 kcal/mol	13.53	94.16	12.01	26.95
$\varepsilon(F)$ =0.088 kcal/mol	12	43.64	14.99	91.65
$\varepsilon(F)$ =0.1 kcal/mol	12.83	49.4	15.02	61.93
$\varepsilon(F)$ =0.113 kcal/mol	13.67	54.87	15.51	54.16
$\varepsilon(F)$ =0.125 kcal/mol	11.78	22.22	20	221.41
$\varepsilon(C_H)$ =0.026 kcal/mol	9.6	92.8	11.03	100.32
$\varepsilon(C_H)$ =0.039 kcal/mol	9.53	52.27	10.25	47.71
$\varepsilon(C_H)$ =0.053 kcal/mol	9.41	33.42	10.32	37.32
$\varepsilon(C_H)$ =0.07 kcal/mol	11.89	61.26	11.39	44.7
$\varepsilon(C_H)$ =0.073 kcal/mol	11.06	46.76	11.17	36.22
$\varepsilon(C_H)$ =0.08 kcal/mol	12.03	60.84	11.49	38.4
$\varepsilon(C_H)$ =0.093 kcal/mol	13.64	96.34	11.82	38.08
$\varepsilon(C_H)$ =0.1 kcal/mol	14.2	94.04	11.5	29.02
$\varepsilon(C_H)$ =0.11 kcal/mol	14.5	81.32	11.9	30.09
$\varepsilon(C_H)$ =0.12 kcal/mol	14.97	84.68	12.66	42.44
$\varepsilon(C_H)$ =0.147 kcal/mol	15.18	41.26	13.88	59.82
$\varepsilon(C_F)$ =0.012 kcal/mol	14.76	831.97	10.78	212.83
$\varepsilon(C_F)$ =0.026 kcal/mol	10.78	212.83	11.18	118.64
$\varepsilon(C_F)$ =0.039 kcal/mol	11.90	126.94	11.06	71.71
$\varepsilon(C_F)$ =0.066 kcal/mol	11.80	71.09	11.14	71.09
$\varepsilon(C_F)$ =0.08 kcal/mol	11.16	49.03	12.06	54.37
$\varepsilon(C_F)$ =0.093 kcal/mol	11.26	43.52	12.60	53.84

continued on next page

Table S10: Activation energies and pre-exponential factors - Extension.

parameter set	$E_A^{C_6H_{14}}$ in kJ/mol	$D_0^{C_6H_{14}}$ in Å^2/ps	$E_A^{C_6F_{14}}$ in kJ/mol	$D_0^{C_6F_{14}}$ in Å^2/ps
$\varepsilon(C_F)$ =0.107 kcal/mol	10.66	27.06	12.61	44.33
$\varepsilon(C_F)$ =0.12 kcal/mol	11.79	42.76	13.41	52.26
$\varepsilon(C_F)$ =0.134 kcal/mol	11.21	26.74	14.71	78.3
$\varepsilon(C_F)$ =0.147 kcal/mol	14.71	78.3	15.48	92.27
$\varepsilon(C_F)$ =0.161 kcal/mol	11.53	25.85	16.65	117.34
$\varepsilon(C_F)$ =0.174 kcal/mol	12.80	40.91	17.33	122.62
$\varepsilon(C_F)$ =0.201 kcal/mol	11.43	18.85	18.27	117.32
$\sigma(H)$ =1.87 Å	9.64	32.2	8.76	11.78
$\sigma(H)$ =2 Å	9.38	25.49	9.91	20.43
$\sigma(H)$ =2.12 Å	9.88	29.01	9.17	13.89
$\sigma(H)$ =2.37 Å	11.33	32.26	9.64	18.72
$\sigma(H)$ =2.5 Å	11.8	63.4	11.47	48.7
$\sigma(H)$ =2.62 Å	11.27	64.83	11.57	57.72
$\sigma(H)$ =2.75 Å	10.88	58.2	10.88	42.93
$\sigma(H)$ =2.87 Å	12.12	123.03	11.18	55.03
$\sigma(H)$ =3 Å	10.82	71.78	12.48	112.25
$\sigma(H)$ =3.12 Å	11.11	90.62	10.47	46.29
$\sigma(H)$ =3.25 Å	11.79	204.32	13.41	223.12
$\sigma(H)$ =3.37 Å	13.73	450.2	13.95	329.67
$\sigma(H)$ =3.5 Å	11.62	180.26	14.58	504.36
$\sigma(H)$ =3.62 Å	11.9	240.54	14.32	511.94
$\sigma(H)$ =3.75 Å	11.86	308.93	14.85	806.17
$\sigma(F)$ =2.45 Å	10.85	36.51	11.96	46.13
$\sigma(F)$ =2.7 Å	10.93	44.39	10.87	30.53
$\sigma(F)$ =2.95 Å	11.8	30.72	11.86	70.45
$\sigma(F)$ =3.2 Å	11.01	65.2	11.11	50.69
$\sigma(F)$ =3.33 Å	12.29	134.42	10.31	37.39
$\sigma(F)$ =3.45 Å	11.27	89.18	10.48	45.91
$\sigma(F)$ =3.58 Å	11.25	100.44	8.53	17.81
$\sigma(F)$ =3.7 Å	13.14	277.22	10.56	59.29
$\sigma(F)$ =3.95 Å	13.79	490.14	10.93	100.44
$\sigma(F)$ =4.2 Å	11.86	231.27	11.51	205.49
$\sigma(C_H)$ =2.63 Å	10.46	274.18	12.24	232.94
$\sigma(C_H)$ =2.75 Å	11.54	322.98	12.87	293.3
$\sigma(C_H)$ =2.88 Å	10.72	143.19	10.64	73.75
$\sigma(C_H)$ =3.13 Å	11.04	129.02	11.43	95.45
$\sigma(C_H)$ =3.25 Å	9.88	97.04	10.13	39.95
$\sigma(C_H)$ =3.38 Å	10.72	44.27	11.41	67.45
$\sigma(C_H)$ =3.5 Å	11.8	55.68	10.15	28.9
$\sigma(C_H)$ =3.63 Å	11.83	66.93	11.44	42.99
$\sigma(C_H)$ =3.75 Å	10.84	36.96	10.56	24.7
$\sigma(C_H)$ =3.88 Å	11.41	45.84	11.38	36.24

continued on next page

Table S10: Activation energies and pre-exponential factors - Extension.

parameter set	$E_A^{C_6H_{14}}$ in kJ/mol	$D_0^{C_6H_{14}}$ in Å^2/ps	$E_A^{C_6F_{14}}$ in kJ/mol	$D_0^{C_6F_{14}}$ in Å^2/ps
$\sigma(C_H)$ =4 Å	10.74	31.78	10.74	25.9
$\sigma(C_H)$ =4.13 Å	10.21	25.17	10.23	19.9
$\sigma(C_H)$ =4.25 Å	10.11	25.37	9.62	14.78
$\sigma(C_H)$ =4.38 Å	9.53	18.51	10.2	20.91
$\sigma(C_H)$ =4.5 Å	10.85	37.32	9.3	12.55
$\sigma(C_F)$ =2.5 Å	15.60	1166.08	13.25	995.95
$\sigma(C_F)$ =2.62 Å	13.64	405.65	12.39	397.94
$\sigma(C_F)$ =2.75 Å	13.80	421.58	13.10	465.96
$\sigma(C_F)$ =3 Å	14.46	802.72	12.43	252.72
$\sigma(C_F)$ =3.13 Å	12.34	534.59	11.08	95.55
$\sigma(C_F)$ =3.25 Å	11.46	152.7	10.96	67.86
$\sigma(C_F)$ =3.37 Å	10.68	89.14	10.84	54.29
$\sigma(C_F)$ =3.5 Å	11.80	49.61	10.96	48.51
$\sigma(C_F)$ =3.62 Å	11.23	52.53	10.94	31.49
$\sigma(C_F)$ =3.75 Å	10.48	31.66	11.79	40.96
$\sigma(C_F)$ =3.87 Å	10.61	29.78	11.67	32.12
$\sigma(C_F)$ =4 Å	11.33	37.31	12.10	27.01
$\sigma(C_F)$ =4.12 Å	10.88	27.01	12.85	42.26
$\sigma(C_F)$ =4.25 Å	10.15	16.63	13.56	19.94
$\sigma(C_F)$ =4.37 Å	10.78	22.51	12.99	36.81
$\sigma(C_F)$ =4.5 Å	11.29	26.24	13.25	39.97
$\sigma(C_F)$ =4.62 Å	11.69	30.84	14.29	66.15

2.2 Pre-exponential factors of diffusion

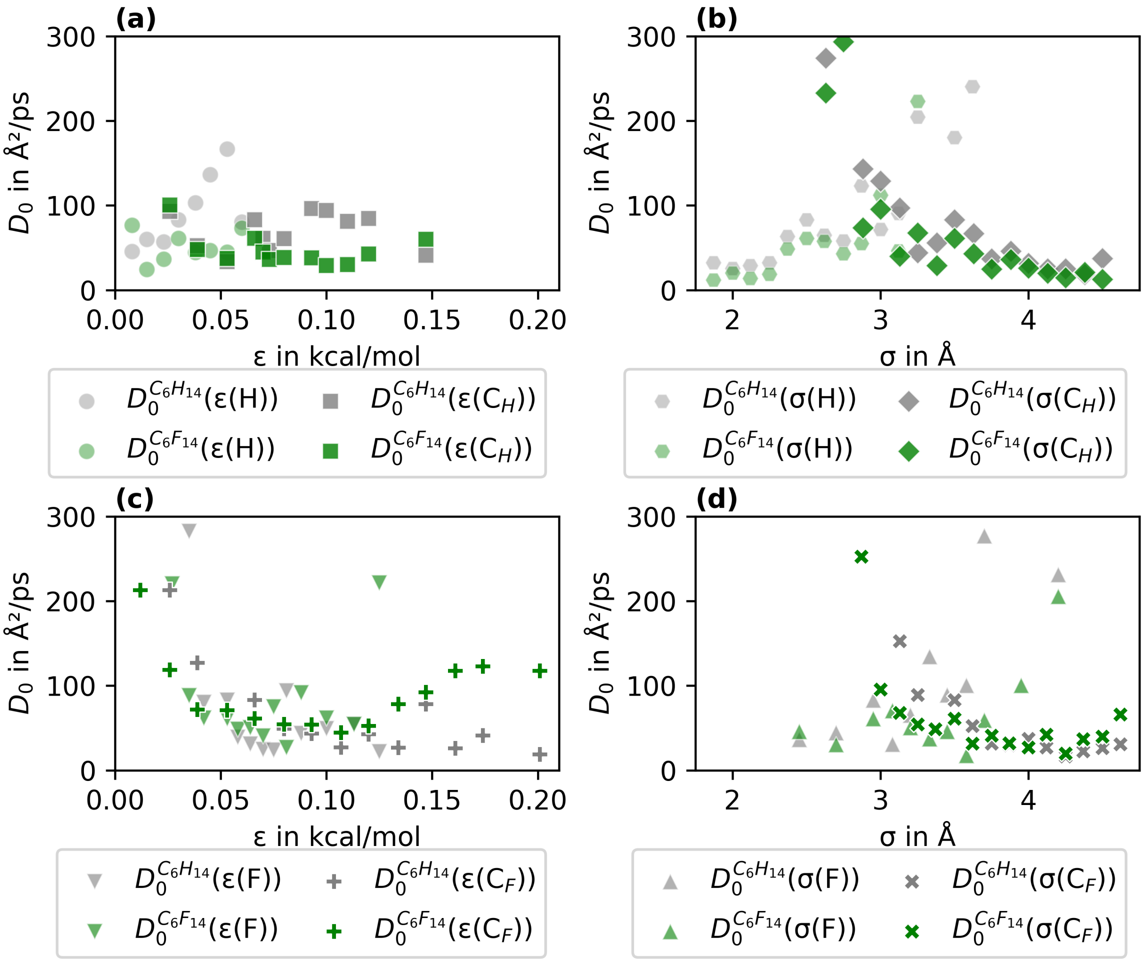

Figure S2: Pre-exponential factors of hexane (grey symbols) and perfluorohexane (green symbols) diffusion in a 1:1 mixture as a function of (a) $\varepsilon(H)$ and $\varepsilon(C_H)$, (b) $\sigma(H)$ and $\sigma(C_H)$, (c) $\varepsilon(F)$ and $\varepsilon(C_F)$, and (d) $\sigma(F)$ and $\sigma(C_F)$. Standard force field (OPLS-AA) values are $\varepsilon(H) = 0.03\,\mathrm{kcal/mol}$[1], $\varepsilon(C_H) = 0.066\,\mathrm{kcal/mol}$[1], $\sigma(H) = 2.5\,\text{Å}$[1], $\sigma(C_H) = 3.5\,\text{Å}$[1], $\varepsilon(F) = 0.053\,\mathrm{kcal/mol}$[2], $\varepsilon(C_F) = 0.066\,\mathrm{kcal/mol}$[2], $\sigma(F) = 2.95\,\text{Å}$[2], and $\sigma(C_F) = 3.5\,\text{Å}$[2].

3 Configurational entropy of mixing

3.1 Additional entropy-time curves

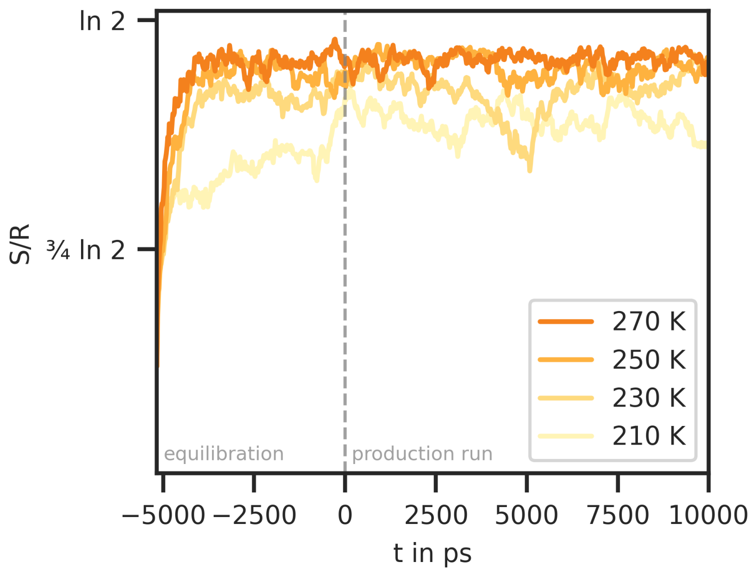

Figure S3: Entropy of mixing of hexane and perfluorohexane during simulations using OPLS-AA force field parameters at different temperatures. Units of R.

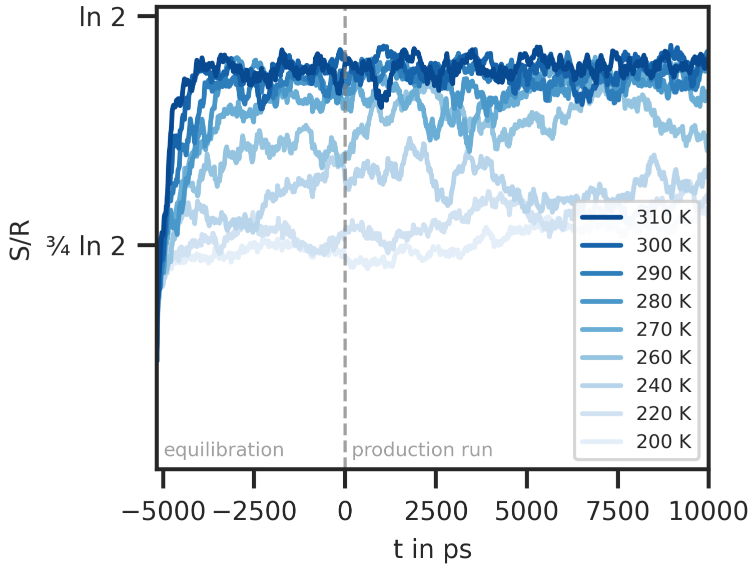

Figure S4: Entropy of mixing of hexane and perfluorohexane during simulations with a modified Lennard-Jones energy parameter of $\varepsilon(F) = 0.1\,\text{kcal/mol}$ at different temperatures. Units of R.

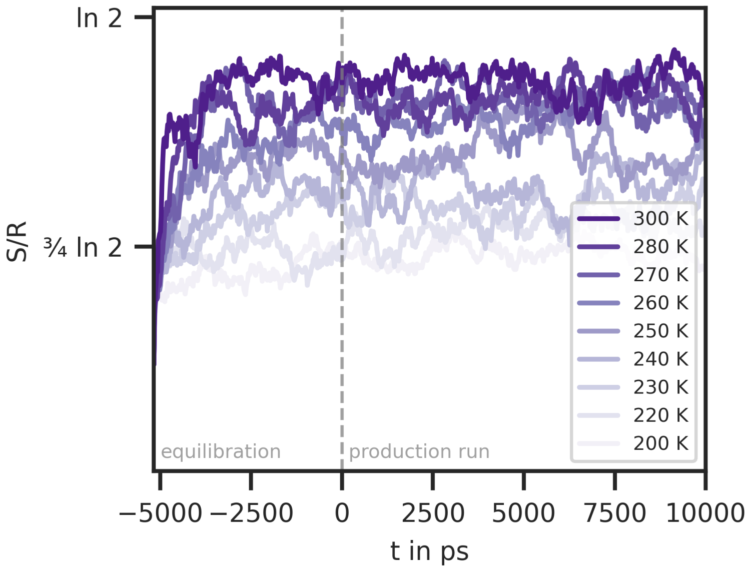

Figure S5: Entropy of mixing of hexane and perfluorohexane during simulations with a modified Lennard-Jones energy parameter of $\varepsilon(C_H) = 0.093\,\mathrm{kcal/mol}$ at different temperatures. Units of R.

3.2 Exponential fitting of the entropy-time curves

All entropy-time curves were fitted with an exponential function according to Equation 2[4].

$$S(t) = S_0 + S\infty\left(1 - \exp\left(-\frac{t}{\tau}\right)\right) \tag{2}$$

Table S11: Fit coefficients from fitting configurational entropy of mixing curves with an exponential function (Equation 2). The converged entropy value is $S_{\text{conv}} = S_0 + S\infty$.

parameter set	T in K	S_0 in 1/R	S_∞ in 1/R	τ in ps	S_{conv} in 1/R
OPLS-AA	200	0.48	0.11	1045	0.59
OPLS-AA	220	0.52	0.11	2740	0.63
OPLS-AA	230	0.46	0.18	450	0.64
OPLS-AA	240	0.45	0.20	380	0.66
OPLS-AA	250	0.46	0.20	424	0.66
OPLS-AA	260	0.43	0.22	309	0.66
OPLS-AA	270	0.47	0.19	275	0.66
OPLS-AA	280	0.44	0.22	184	0.66
OPLS-AA	300	0.42	0.24	188	0.66
$\varepsilon(F) = 0.1$ kcal/mol	200	0.44	0.07	74	0.51
$\varepsilon(F) = 0.1$ kcal/mol	220	0.49	0.04	867	0.53
$\varepsilon(F) = 0.1$ kcal/mol	240	0.50	0.08	2456	0.58
$\varepsilon(F) = 0.1$ kcal/mol	260	0.49	0.12	1460	0.61
$\varepsilon(F) = 0.1$ kcal/mol	270	0.47	0.16	716	0.63
$\varepsilon(F) = 0.1$ kcal/mol	280	0.46	0.19	748	0.64
$\varepsilon(F) = 0.1$ kcal/mol	290	0.47	0.18	608	0.65
$\varepsilon(F) = 0.1$ kcal/mol	300	0.45	0.20	406	0.65
$\varepsilon(F) = 0.1$ kcal/mol	310	0.45	0.20	288	0.65
$\varepsilon(C_H) = 0.093$ kcal/mol	200	0.48	0.04	3785	0.52
$\varepsilon(C_H) = 0.093$ kcal/mol	220	0.46	0.07	187	0.52
$\varepsilon(C_H) = 0.093$ kcal/mol	230	0.48	0.07	1189	0.55
$\varepsilon(C_H) = 0.093$ kcal/mol	240	0.47	0.09	833	0.57
$\varepsilon(C_H) = 0.093$ kcal/mol	250	0.47	0.12	551	0.59
$\varepsilon(C_H) = 0.093$ kcal/mol	260	0.48	0.14	892	0.61
$\varepsilon(C_H) = 0.093$ kcal/mol	270	0.46	0.17	629	0.63
$\varepsilon(C_H) = 0.093$ kcal/mol	280	0.44	0.19	380	0.63
$\varepsilon(C_H) = 0.093$ kcal/mol	300	0.50	0.15	518	0.65

3.3 Entropy of mixing as a function of system size

In our mixing/demixing simulations, we observe that the transition from separate to mixed configurations does not occur instantly, but rather spans a wide temperature range around 40 K. This range is clearly too large for the "true" (experimental) phase transition. We expect it to narrow with increasing simulation time and box size. To test which constraint is more relevant, we performed an additional simulation with OPLS force field parameters at T = 220 K with a box size eight times larger than the original (2x2x2). We computed the resulting entropy evolution in addition to Figure 6 of the manuscript. The results are shown in Figure S6.

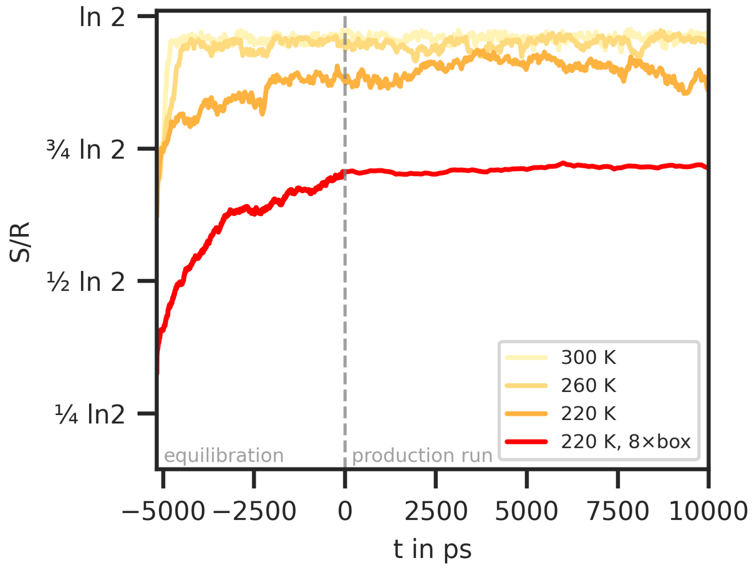

Figure S6: Entropy of mixing of hexane and perfluorohexane during simulations using OPLS-AA force field parameters at 220 K with different box sizes. Entropy curves at 260 K and 300 K are given for comparison. Units of R.

First, the configurational entropy of mixing clearly converges after 10 ns. The evolution is practically flat, indicating that, for system sizes of less than a 15 nm box length, finite simulation time is not a decisive factor. However, this may change when increasing the system size to macroscopic dimensions, such as micrometer or millimeter length scales, where equilibration times will be orders of magnitude larger.

Second, the numerical entropy value of the 2x2x2 system is significantly lower than that of the 1x1x1 system, which may seem surprising at first. This behavior occurs because of the periodic boundary conditions. Even a system that is fully phase-separated into two layers A and B within one simulation box appears well-mixed when viewed from a larger perspective (e.g., when considering a 4x4x4 periodic replication of the original box). From this larger perspective, the system appears multilamellar (A|B|A|B|A|B|A|B), and it can still become more phase-separated (AAAA|BBBB). Hence, the entropy of mixing for this system should not approach zero but rather an intermediate value. This effect weakens with increasing system size, which was analyzed in more detail in a previous publication [5]. Thus, the entropy value of the phase-separated 2x2x2 system will be closer to zero than the 1x1x1 system.

In conclusion, this analysis confirms that our simulation results indicate a gradual transition from mixed to phase-separated, which we however attribute to the combined effect of size and simulation time limitations.

References

[1] W. L. Jorgensen, D. S. Maxwell, J. Tirado-Rives, *J. Am. Chem. Soc.* **1996**, *118*, 11225.

[2] E. K. Watkins, W. L. Jorgensen, *J. Phys. Chem. A* **2001**, *105*, 4118.

[3] P. Morgado, L. F. G. Martins, E. J. M. Filipe, *Phys. Chem. Chem. Phys.* **2019**, *21*, 3742.

[4] T. Hanke, A. L. Upterworth, D. Sebastiani, *J. Phys. Chem. Lett.* **2024**, *15*, 11320.

[5] T. Hanke, D. Sebastiani, *J. Chem. Phys.* **2026**, *164*, 034101.
